# The immunogenicity and safety of an inactivated quadrivalent influenza vaccine and a 23-valent pneumococcal polysaccharide vaccine in individuals with chronic diseases

**DOI:** 10.3389/fimmu.2025.1624095

**Published:** 2025-08-01

**Authors:** Wanqin Tang, Xi Lu, Zhongkui Zhu, Dan Yu, Huaxian Liu, Yufei Song, Lu Shen, Yang Yu, Yanwei Zhao, Yan Xie

**Affiliations:** ^1^ Department of Expanded Programme on Immunization, Taizhou City Center for Disease Control and Prevention, Taizhou, Jiangsu, China; ^2^ Clinical Research and Development Center, Sinovac Biotech Co., Ltd., Beijing, China

**Keywords:** chronic disease, inactivated quadrivalent influenza vaccine, 23-valent pneumococcal polysaccharide vaccine, simultaneous administration, immunogenicity, safety

## Abstract

**Background:**

The inactivated quadrivalent influenza vaccine (IIV4) and the 23-valent pneumococcal polysaccharide vaccine (PPSV23) are widely administered. However, there was limited clinical evidence on the immunogenicity and safety of administration of the two vaccines in individuals with chronic diseases, especially concerning simultaneous administration.

**Methods:**

A total of 480 participants aged ≥60 years were randomly assigned to receive simultaneous or separate administration of IIV4 and PPSV23 and categorized into the Chronic Disease group or Healthy group based on their baseline health status. Blood samples were drawn before and 28 days after each vaccination to test the antibodies against all four influenza virus strains and 23 pneumococcus serotypes.

**Results:**

The geometric mean titer ratios (Chronic Disease group/Healthy group) of influenza antibodies ranged from 1.04 to 1.37 in the whole population and from 1.02 to 1.39 in the simultaneous administration population. The geometric mean concentration ratios of pneumococcal antibodies ranged from 0.87 to 1.12 in the whole population and from 0.97 to 1.33 in the simultaneous administration population. All ratios met the criteria for non-inferiority. The rate of adverse events was 0.96% in the Chronic Disease group and 1.47% in the Healthy group, with most events being mild (grade 1). No serious adverse events were observed.

**Conclusion:**

The immunogenicity and safety profiles of IIV4 and PPSV23, particularly when administered simultaneously, in individuals with chronic diseases were comparable to those in healthy individuals, supporting the vaccination strategy of IIV4 and PPSV23 in chronic disease population.

## 1 Introduction

Individuals with chronic diseases face a higher risk of complications than healthy individuals when infected with infectious diseases, such as influenza and pneumonia, which could lead to prolonged illness, hospitalization, and even death (1, 2). Individuals with diabetes, hypertension, and other chronic diseases exhibit significantly higher rates of influenza-related hospitalizations and excess mortality compared to healthy individuals (3–5). These heightened risks have also been observed consistently in pneumococcal infections (6, 7).

Multiple studies have demonstrated the benefits of administering seasonal influenza vaccines and pneumococcal polysaccharide vaccines to individuals with chronic diseases (8–10). Influenza vaccination was well-tolerated and significantly reduced the hospitalization and mortality rates among diabetic patients (11, 12). Influenza vaccination could also lower the risk of chronic kidney disease (CKD) in hypertensive patients and reduce the need for kidney replacement therapy (13, 14). This potential protective effect could persist during both influenza and non-influenza seasons. The 23-valent pneumococcal polysaccharide vaccine (PPSV23) vaccination could reduce the disease burden of invasive pneumococcal diseases (15, 16) and demonstrate significant cost-effectiveness (17) among older individuals and individuals with underlying conditions such as chronic obstructive pulmonary disease (COPD), diabetes, and heart disease. Administering both influenza vaccines and PPSV23 to COPD patients can reduce pneumonia-related hospitalizations by 63% and overall mortality by 81% (18).

However, there was limited clinical evidence on the immunogenicity and safety profiles of these two vaccines in chronic disease population, especially on simultaneous administration in this population. Therefore, this clinical trial aimed to evaluate these profiles in individuals with chronic diseases that were not contraindicated for vaccination, and to provide evidence for developing vaccination strategies, particularly regarding simultaneous administration, in chronic disease populations.

## 2 Materials and methods

### 2.1 Study design and participants

This phase IV, controlled, open-label, non-inferiority trial (ClinicalTrials.gov ID: NCT05471531) was conducted by Taizhou City Center for Disease Control and Prevention to evaluate the immunogenicity and safety profiles of quadrivalent inactivated influenza vaccine (IIV4) and PPSV23 in individuals with chronic diseases based on our previous study (19).

Participants (aged ≥60 years) who were able to comprehend and sign informed consent were screened. The key exclusion criteria were participants who (1) had received the 2021–2022 seasonal influenza vaccine prior to the screening; (2) had received PPSV23 within the past five years; (3) had a documented history of severe allergic reactions to the two vaccines; (4) had been diagnosed with uncontrolled epilepsy or other severe neurological condition; (5) were currently suffering from fever, acute exacerbation of a chronic condition, uncontrolled serious chronic disease or acute illness; (6) had any additional risk factors that the investigator considered unsuitable for participation. Detailed inclusion and exclusion criteria are provided in the **Supplementary Materials**.

All essential documents for this study received ethical approval from the Ethics Committee of Taizhou City Hospital of Traditional Chinese and Western Medicine (approval number: 2021-Ethics Review-11). All procedures were conducted in accordance with the Declaration of Helsinki and Good Clinical Practice (GCP) guidelines issued by the International Council for Harmonization (ICH). Written informed consent was obtained from all participants prior to screening.

### 2.2 Investigational vaccines

The IIV4 and PPSV23 were both developed and produced by Sinovac Biotech Co., Ltd.,(Beijing, China). Each 0.5 mL dose of the IIV4 contained 60 µg of hemagglutinin, 15 µg per strain, covering four WHO-recommended influenza virus strains for the 2021–2022 season: A/Victoria/2570/2019 (H1N1) pdm09-like virus, A/Cambodia/e0826360/2020 (H3N2)-like virus, B/Washington/02/2019 (B/Victoria lineage)-like virus, and B/Phuket/2027/2013 (B/Yamagata lineage)-like virus. Each 0.5 mL dose of PPSV23 contained 25 µg of capsular polysaccharides for each of the 23 serotypes (1, 2, 3, 4, 5, 6B, 7F, 8, 9N, 9V, 10A, 11A, 12F, 14, 15B, 17F, 18C, 19A, 19F, 20, 22F, 23F, and 33F).

### 2.3 Procedures

A total of 480 participants aged ≥60 years were enrolled and randomly assigned to receive simultaneous or separate administration of IIV4 in the right arm and PPSV23 in the left arm. In the simultaneous administration population, 160 participants received a dose of IIV4 and a dose of PPSV23 on day 0. In the separate administration population, 160 participants received a dose of IIV4 on day 0 and a dose of PPSV23 on day 28 (separate administration population 1, S1) and 160 participants received a dose of PPSV23 on day 0 and a dose of IIV4 on day 28 (separate administration population 2, S2). All participants were categorized into the Chronic Disease group (C group) or Healthy group (H group) based on their baseline health status. Blood samples were drawn before and 28 days after each vaccination. Antibodies against the four influenza virus strains and the 23 pneumococcal serotypes were assessed. For safety monitoring, participants were instructed to report all adverse events that occurred within 28 days after each vaccination. Serious adverse events were recorded throughout the trial. Adverse events were graded based on the guidelines of the China National Medical Products Administration (NMPA) (20).

### 2.4 Statistical analysis

#### 2.4.1 Sample size

The primary goal was to determine whether the immunogenicity of IIV4 in the C group was non-inferior to that in the H group as assessed by antibody geometric mean titers (GMTs) against the four influenza virus strains at 28 days following vaccination. The secondary goal was to determine whether the immunogenicity of PPSV23 in the C group was non-inferior to that in the H group, as assessed by antibody geometric mean concentrations (GMCs) against the 23 pneumococcal serotypes at 28 days following vaccination. To evaluate the non-inferiority, the lower limits of the 95% confidence intervals (CIs) for the GMT ratios (GMT_C group_/GMT_H group_) needed to exceed 0.5 (21). This equates to the lower limits of the 95% CIs for the log-transformed GMT differences exceed −0.301. Assuming N _C group_: N _H group_ = 1:1, a one-sided α = 0.025, an overall power of 80%, and the standard deviation after logarithmic transformation was 0.45, the required effective sample size for each group was calculated as 60 participants using the NCSS-PASS software (version: 15.0.5). Considering a 10% dropout rate, each group was planned to recruit 67 participants. And considering the goals of the previous study (19), the final participants were 480 in total.

#### 2.4.2 Immunogenicity assessment

Antibody GMTs, GMCs, geometric mean fold rises (GMFR) and the corresponding 95% CIs were calculated for each group, and differences between groups were tested using analysis of variance (ANOVA) on log-transformed values. The seroconversion rates (SCR) and seroprotection rates (SPR) of IIV4, the 2-fold increase rate of PPSV23, and the corresponding 95% CIs were calculated and differences between groups were tested using the χ² tests or Fisher’s exact test.

Non-inferiority comparisons were analyzed using the analysis of covariance (ANCOVA). Logarithmically transformed post-vaccination antibody GMTs and GMCs were defined as the dependent variable and logarithmically transformed pre-vaccination antibody GMTs or GMCs were defined as the covariate. The least-squares means and 95% CIs for the GMT or GMC ratios between groups were calculated. Non-inferiority was demonstrated if the lower limit of the 95% CI for the GMT or GMC ratio (C group/H group) was greater than 0.5 (21).

The SPR for influenza hemagglutination inhibition (HI) antibodies was characterized as the percentage of participants with an HI titer ≥1:40. The SCR was characterized as the percentage of participants with either a baseline HI titer < 1:10 and a post-vaccination HI titer ≥ 1:40, or a baseline HI titer ≥1:10 and a ≥ 4-fold increase after vaccination (22). The 2-fold increase rate for PPSV23 was characterized as the percentage of participants with at least a 2-fold rise in post-vaccination antibody concentration compare to baseline.

All immunogenicity assessments were performed in the per-protocol set, defined as participants who completed scheduled vaccinations, provided valid serological values at all time points, and demonstrated full compliance without protocol deviations.

#### 2.4.3 Safety assessment

Safety assessments included the recording of adverse events within 28 days following IIV4 and PPSV23 vaccination, and monitoring of serious adverse events throughout the trial. The severity of adverse event was graded according to the National Medical Products Administration (NMPA) guideline (20). The rates and severity grading were assessed in the safety set, which included all participants who received at least one dose of the vaccines. Participants who received the erroneous vaccine were analyzed according to the vaccines they actually received in adherence to the All Subjects as Treated (ASaT) principle. Intergroup comparisons were assessed using Fisher’s exact test.

### 2.5 Serological assays

Serological analyses were performed using standardized methodologies aligned with WHO technical specifications. The antibodies against four influenza vaccine-associated strains (A/Victoria/2570/2019 (H1N1) pdm09-like virus; A/Cambodia/e0826360/2020 (H3N2)-like virus; B/Washington/02/2019 (B/Victoria lineage)-like virus; B/Phuket/2027/2013 (B/Yamagatalineage)-like virus)) were quantified through standardized HI assays performed in strict accordance with the WHO Manual for Laboratory Diagnosis and Virological Surveillance of Influenza (23). The international certified reference standards of influenza antigens were purchased from the National Institute for Biological Standards and Control (NIBSC, London, UK). Pneumococcal serotype-specific IgG antibody concentrations against 23 vaccine-related serotypes were quantified using WHO-validated quantitative enzyme linked immunosorbent assay (ELISA) protocols as detailed in the WHO Manual for Pneumococcal Polysaccharide ELISA (24). Certified pneumococcal polysaccharide standards were purchased from the American Type Culture Collection (ATCC, Manassas, VA, USA).

## 3 Results

### 3.1 Study population

**Figure 1:**
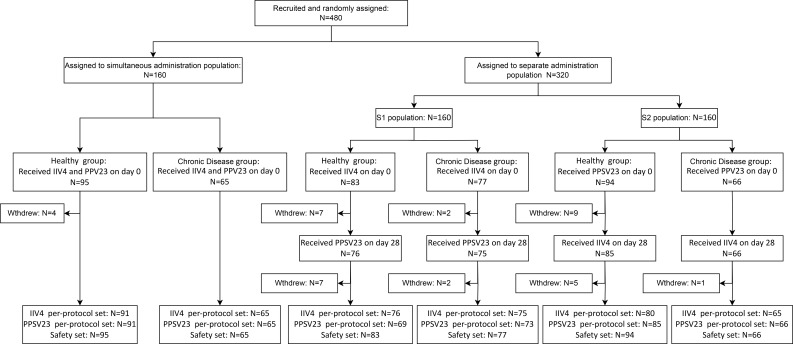
Flowchart of participants’ disposition.

A total of 480 participants were recruited in this clinical trial, 43.33% of whom had pre-existing chronic diseases, primarily hypertension (27.29%), obesity (7.92%), and diabetes (5.00%) (**Supplementary Table S1**). The participants were randomly assigned to the simultaneous vaccination population (95 healthy individuals, 65 individuals with chronic diseases), the separate administration population S1 (83 healthy individuals, 77 individuals with chronic diseases), and the separate administration population S2 (94 healthy individuals, 66 individuals with chronic diseases). And they were categorized into the Chronic Disease group (C group) or Healthy group (H group) based on their baseline health status. In the simultaneous vaccination population, participants received 1 dose of IIV4 and 1 dose of PPSV23 simultaneously on day 0. In the separate administration population S1, participants received 1 dose of IIV4 on day 0 and 1 dose of PPSV23 on day 28. In the separate administration population S2, participants received 1 dose of PPSV23 on day 0 and 1 dose of IIV4 on day 28 (**Figure 1**).

### 3.2 Demographic characteristics

The mean age of participants in the Healthy group (H group) and Chronic Disease group (C group) were 68.46 ± 5.18 years and 68.76 ± 5.52 years, respectively (**Table 1**). All participants were of Han ethnicity. The gender ratios and mean height in the two groups were similar. The mean weight in the H group were lower than that in the C group (p=0.0020). Similarly, except for the difference in mean weight, there were no differences between the Health subgroup (H subgroup) and the Chronic Disease subgroup (C subgroup) in the simultaneous administration population.

**Table 1:** Demographic characteristics and health conditions of participants.

Population	Characteristics		H group or subgroup	C group or subgroup	Total	*P*-value
All	N		272	208	480	
	Age, years *		68.46 ± 5.18	68.76 ± 5.52	68.59 ± 5.33	0.5351
	Age groups, years	60-69, n (%)	175 (64.34)	123 (59.13)	298 (62.08)	0.2781
		70-79, n (%)	87 (31.99)	78 (37.50)	165 (34.38)	
		80-89, n (%)	10 (3.68)	7 (3.37)	17 (3.54)	
	Gender	Male, n (%)	132 (48.53)	96 (46.15)	228 (47.50)	0.6055
		Female, n (%)	140 (51.47)	112 (53.85)	252 (52.50)	
	Ethnicity	Han, n (%)	272 (100.00)	208 (100.00)	480 (100.00)	1.0000
	Height, cm*		162.05 ± 8.43	161.17 ± 8.61	161.67 ± 8.51	0.2618
	Weight, kg*		63.70 ± 9.31	67.82 ± 12.12	65.49 ± 10.80	<0.0001
Simultaneous population**	N		95	65	160	
	Age, years *		68.55 ± 5.37	68.09 ± 5.22	68.36 ± 5.30	0.5952
	Age groups, years	60-69, n (%)	64 (67.37)	42 (64.62)	106 (66.25)	0.8201
		70-79, n (%)	27 (28.42)	22 (33.85)	49 (30.63)	
		80-89, n (%)	4 (4.21)	1 (1.54)	5 (3.13)	
	Gender	Male, n (%)	40 (42.11)	34 (52.31)	74 (46.25)	0.2037
		Female, n (%)	55 (57.89)	31 (47.69)	86 (53.75)	
	Ethnicity	Han, n (%)	95 (100.00)	65 (100.00)	160 (100.00)	1.0000
	Height, cm*		162.09 ± 8.56	162.23 ± 8.15	162.15 ± 8.37	0.9200
	Weight, kg*		63.46 ± 9.30	69.48 ± 13.22	65.91 ± 11.41	0.0020

*Mean ± standard deviation; **simultaneous population, simultaneous administration population; H group or subgroup, Healthy group or subgroup; C group or subgroup, chronic disease group or subgroup.

### 3.3 Immunogenicity

#### 3.3.1 Immunogenicity of IIV4 in the chronic disease group and the healthy group

Before IIV4 vaccination, GMTs of HI antibodies against four influenza virus strains in the H group and the C group ranged from 11.90 to 61.11 and from 12.89 to 75.79, respectively (**Table 2**). The SPRs in the two groups ranged from 14.57% to 78.95% and from 17.07% to 88.78%, respectively. For the A/H3N2 strain, the GMT and SPR in the C group were higher than those in the H group (p=0.0051 and p=0.0102, respectively). For the other three strains, there were no differences in GMTs and SPRs between the two groups.

**Table 2:** Pre-vaccination antibodies against four influenza virus strains.

Population	Strain	Variable	H group or subgroup		C group or subgroup		Total		*P*-value
			Value	95%CI	Value	95%CI	Value	95%CI	
All	N		247		205		452		
	A/H1N1	SPR, % (n)	21.05 (52)	16.14,26.67	21.46 (44)	16.05,27.72	21.24 (96)	17.56-25.30	0.9153
		GMT	11.90	10.42,13.59	12.89	10.99,15.11	12.34	11.14-13.66	0.4459
	A/H3N2	SPR, % (n)	78.95 (195)	73.33,83.86	88.78 (182)	83.64,92.75	83.41 (377)	79.65-86.72	0.0051
		GMT	61.11	54.46,68.57	75.79	67.52,85.07	67.37	62.06-73.15	0.0102
	B/Victoria	SPR, % (n)	14.57 (36)	10.42,19.60	17.07 (35)	12.19,22.94	15.71 (71)	12.48-19.40	0.4674
		GMT	12.95	11.71,14.32	13.56	12.09,15.20	13.22	12.26-14.25	0.5501
	B/Yamagata	SPR, % (n)	53.04 (131)	46.61,59.39	51.71 (106)	44.64,58.72	52.43 (237)	47.72-57.12	0.7782
		GMT	32.96	29.75,36.51	31.57	28.46,35.01	32.32	30.05-34.76	0.5635
Simultaneous population*	N		91		65		156		
	A/H1N1	SPR, % (n)	21.98 (20)	13.97,31.88	23.08 (15)	13.53,35.19	22.44 (35)	16.15,29.80	0.8711
		GMT	12.96	10.33,16.25	12.38	9.42,16.26	12.71	10.70,15.10	0.7972
	A/H3N2	SPR, % (n)	76.92 (70)	66.91,85.11	89.23 (58)	79.06,95.56	82.05 (128)	75.11,87.73	0.0483
		GMT	56.35	46.41,68.43	65.33	53.82,79.29	59.93	52.20,68.81	0.2988
	B/Victoria	SPR, % (n)	16.48 (15)	9.53,25.73	7.69 (5)	2.54,17.05	12.82 (20)	8.01,19.10	0.1054
		GMT	13.26	11.17,15.72	13.92	11.85,16.35	13.53	12.01,15.24	0.6796
	B/Yamagata	SPR, % (n)	49.45 (45)	38.80,60.14	47.69 (31)	35.15,60.46	48.72 (76)	40.65,56.84	0.8285
		GMT	28.83	24.64,33.73	29.05	24.33,34.68	28.92	25.74,32.49	0.9494

*Simultaneous population, simultaneous administration population. H group or subgroup, Healthy group or subgroup; C group or subgroup, chronic disease group or subgroup. SPR, seroprotection rate (≥1:40); GMT, geometric mean titer.

**Figure 2:**
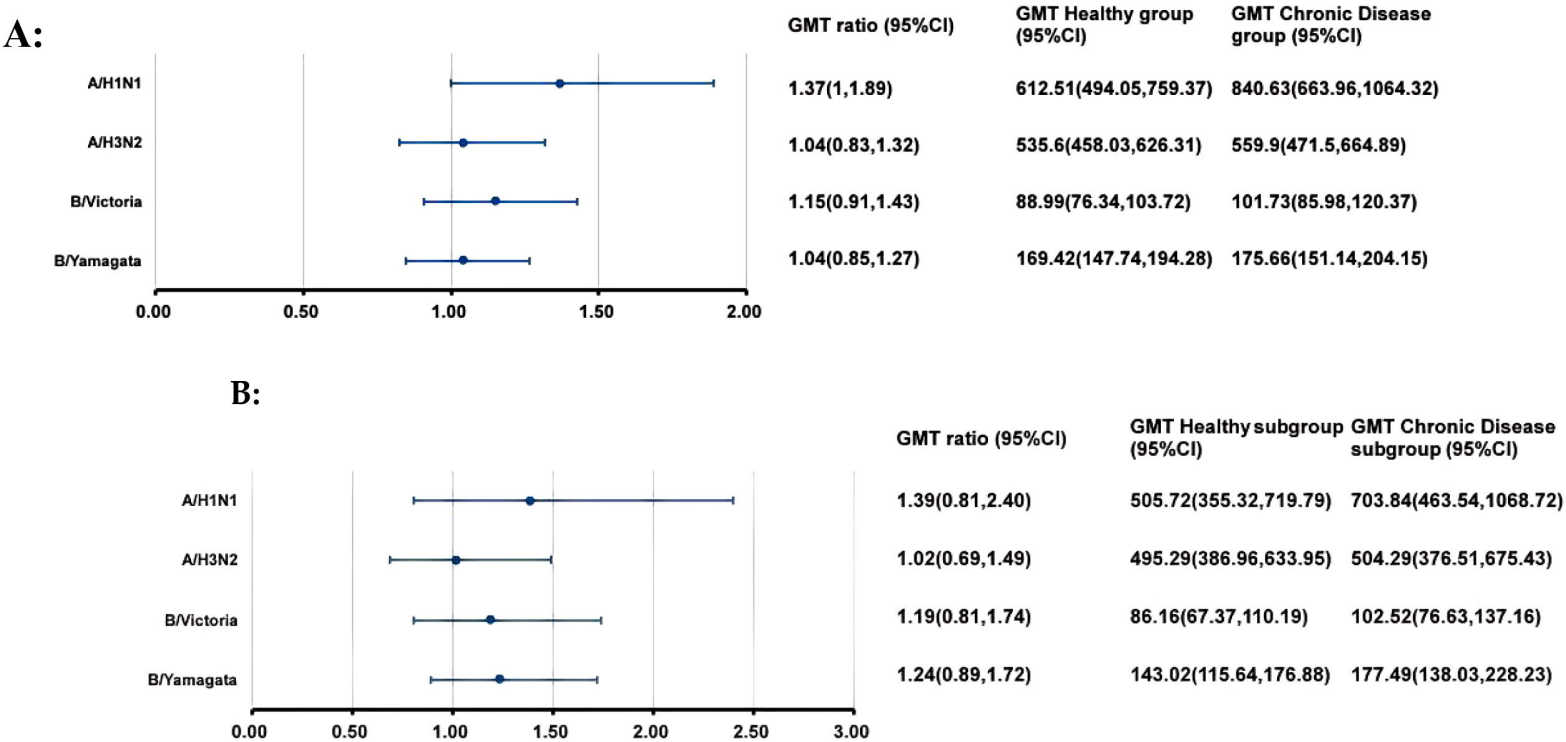
GMTs and GMT ratios of influenza antibodies against four influenza virus strains after IIV4 vaccination. At 28 days after IIV4 vaccination, the antibody GMTs, GMT ratios (Chronic Disease group/ Healthy group), and 95% CIs were calculated. To evaluate the non-inferiority, the lower limits of the 95% CIs for the GMT ratios needed to exceed 0.5. **(A)** the entire clinical trial population; **(B)** simultaneous administration population. GMT, geometric mean titer; HI, hemagglutination inhibition; IIV4, inactivated quadrivalent influenza vaccine; CI, confidence interval.

At 28 days after IIV4 vaccination, the ANCOVA adjusted GMTs of HI antibodies against the four influenza virus strains showed significant increases, ranging from 88.99 to 612.51 in the H group and from 101.73 to 840.63 in the C group (**Figure 2A**). The GMT ratios of the C group to the H group (C group/H group) ranged from 1.04 to 1.37, all the lower limits of the 95% CIs (0.83-1.00) exceeded the non-inferiority thresholds. The SPRs, SCRs and GMFRs in both the H group and the C group reached >80%, >70% and >5-fold, respectively, with all values meeting the immunogenicity criteria of European Medicines Agency for influenza vaccines (25) (**Table 3**). And there were no differences in SPRs, SCRs, and GMFRs between the two groups.

**Table 3:** Post-vaccination antibodies against four influenza virus strains.

Population	Strain	Variable	H group or subgroup		C group or subgroup		Total		*P*-value
			Value	95%CI	Value	95%CI	Value	95%CI	
All	N		247		205		452		
	A/H1N1	SPR, % (n)	90.69 (224)	86.36,94.01	94.15 (193)	90.00,96.94	92.26 (417)	89.40-94.55	0.1709
		SCR, % (n)	89.07 (220)	84.50,92.67	93.17 (191)	88.81,96.22	90.93 (411)	87.90-93.41	0.1306
		GMFR	50.14	40.19,62.54	67.33	53.15,85.29	57.31	48.76-67.35	0.0740
	A/H3N2	SPR, % (n)	97.98 (242)	95.34,99.34	99.51 (204)	97.31,99.99	98.67 (446)	97.13-99.51	0.2278
		SCR, % (n)	76.11 (188)	70.30,81.29	76.10 (156)	69.66,81.76	76.11 (344)	71.90-79.97	0.9969
		GMFR	8.56	7.14,10.26	7.60	6.30,9.18	8.11	7.12-9.24	0.3769
	B/Victoria	SPR, % (n)	82.59 (204)	77.28,87.11	83.41 (171)	77.60,88.23	82.96 (375)	79.18-86.32	0.8166
		SCR, % (n)	72.47 (179)	66.45,77.94	73.66 (151)	67.07,79.55	73.01 (330)	68.66-77.05	0.7768
		GMFR	6.80	5.80,7.97	7.60	6.36,9.10	7.15	6.35-8.05	0.3559
	B/Yamagata	SPR, % (n)	94.33 (233)	90.67,96.87	96.59 (198)	93.09,98.62	95.35 (431)	92.99-97.10	0.2572
		SCR, % (n)	70.85 (175)	64.75,76.44	70.73 (145)	63.99,76.86	70.8 (320)	66.37-74.95	0.9780
		GMFR	5.18	4.44,6.04	5.52	4.69,6.48	5.33	4.77-5.95	0.5782
Simultaneous population*	N		91		65		156		
	A/H1N1	SPR, % (n)	92.31 (84)	84.79,96.85	90.77 (59)	80.98,96.54	91.67 (143)	86.17,95.49	0.7318
		SCR, % (n)	90.11 (82)	82.05,95.38	89.23 (58)	79.06,95.56	89.74 (140)	83.88,94.02	0.8584
		GMFR	39.61	27.27,57.52	55.72	37.41,82.99	45.66	34.78,59.94	0.2231
	A/H3N2	SPR, % (n)	100.00 (91)	96.03,100.00	100.00 (65)	94.48,100.00	100.00 (156)	97.66,100.00	1.0000
		SCR, % (n)	75.82 (69)	65.72,84.19	78.46 (51)	66.51,87.69	76.92 (120)	69.51,83.28	0.6999
		GMFR	8.63	6.49,11.48	7.92	5.74,10.91	8.33	6.74,10.28	0.6900
	B/Victoria	SPR, % (n)	81.32 (74)	71.78,88.72	86.15 (56)	75.34,93.47	83.33 (130)	76.54,88.81	0.4243
		SCR, % (n)	68.13 (62)	57.53,77.51	78.46 (51)	66.51,87.69	72.44 (113)	64.72,79.28	0.1546
		GMFR	6.41	4.92,8.36	7.50	5.67,9.92	6.85	5.65,8.29	0.4267
	B/Yamagata	SPR, % (n)	91.21 (83)	83.41,96.13	98.46 (64)	91.72,99.96	94.23 (147)	89.33,97.33	0.0810
		SCR, % (n)	67.03 (61)	56.39,76.53	73.85 (48)	61.46,83.97	69.87 (109)	62.02,76.95	0.3605
		GMFR	4.95	3.98,6.16	6.13	4.71,7.97	5.41	4.58,6.40	0.2151

*Simultaneous population, simultaneous administration population. H group or subgroup, Healthy group or subgroup; C group or subgroup, chronic disease group or subgroup. SPR, seroprotection rate (≥1:40); SCR, seroconversion rate; GMFR, geometric mean fold rise.

#### 3.3.2 Immunogenicity of IIV4 in the chronic disease subgroup and the healthy subgroup after simultaneous vaccination

In the simultaneous vaccination population, the pre-vaccination SPR against the A/H3N2 strain in the C subgroup was higher than that in the H subgroup (p=0.0483) (**Table 2**). No differences were observed in GMTs and other SPRs between the two subgroups.

At 28 days after simultaneous vaccination, the ANCOVA adjusted GMTs of HI antibodies against the four influenza virus strains in the H subgroup and C subgroup ranged from 86.16 to 505.72 and from 102.52 to 703.84, respectively (**Figure 2B**). The GMT ratios (C subgroup/H subgroup) ranged from 1.02 to 1.39. All lower limits of the 95% CIs exceeded the non-inferiority thresholds. And there were no differences in post-vaccination SPRs, SCRs, and GMFRs between the two subgroups, with all values meeting the immunogenicity criteria of European Medicines Agency for influenza vaccines (**Table 3**).

#### 3.3.3 Immunogenicity of IIV4 in the chronic disease subgroup and the healthy subgroup after separate vaccination

In the separate vaccination population, the pre-vaccination GMT and SPR against the A/H3N2 strain in the C subgroup were higher than those in the H subgroup (p=0.0472 and p=0.0223, respectively) (**Table 4**). For the other three strains, there were no differences in GMTs and SPRs between the two groups.

**Table 4:** Pre-vaccination antibodies level of four influenza virus strains in the separate administration population.

Strain	Variable	H subgroup		C subgroup		Total		P value
		(N=156)		(N=140)		(N=296)		
		Value	95%CI	Value	95%CI	Value	95%CI	
A/H1N1	SPR, % (n)	20.51 (32)	14.47,27.71	20.71 (29)	14.33,28.38	20.61 (61)	16.15,25.67	0.9659
	GMT	11.32	9.60,13.36	13.13	10.77,16.00	12.15	10.69,13.79	0.2541
A/H3N2	SPR, % (n)	80.13 (125)	73.00,86.08	88.57 (124)	82.10,93.32	84.12 (249)	79.45,88.09	0.0472
	GMT	64.06	55.46,74.00	81.20	70.35,93.71	71.66	64.70,79.37	0.0223
B/Victoria	SPR, % (n)	13.46 (21)	8.53,19.84	21.43 (30)	14.95,29.16	17.23 (51)	13.11,22.02	0.0700
	GMT	12.77	11.26,14.48	14.44	11.75,17.75	13.33	11.96,14.85	0.2876
B/Yamagata	SPR, % (n)	55.13 (86)	46.97,63.09	53.57 (75)	44.95,62.03	54.39 (161)	48.53,60.17	0.7883
	GMT	35.64	31.18,40.72	32.81	28.86,37.31	34.27	31.24,37.59	0.3815

**Figure 3:**
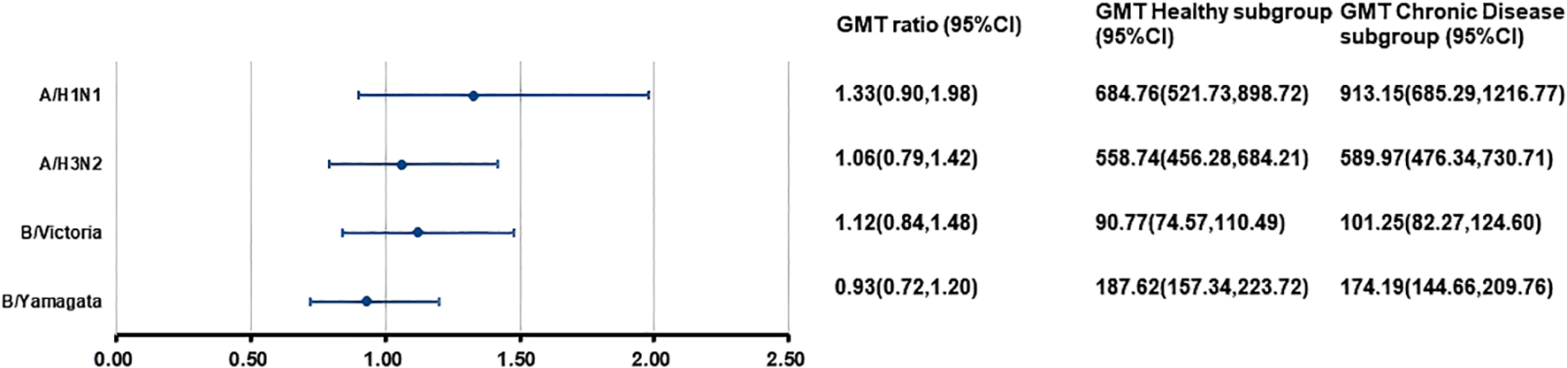
Post-vaccination GMTs and GMT ratios for HI antibodies against four influenza virus strains in the separate administration population. At 28 days after vaccination with IIV4, the HI antibody GMTs, GMT ratios (GMT Chronic Disease subgroup/GMT Healthy subgroup), and 95%CIs were calculated. GMT, adjusted geometric mean titer; HI, hemagglutination inhibition; IIV4, inactivated quadrivalent influenza vaccine; CI, confidence interval.

At 28 days after separate vaccination, the ANCOVA adjusted GMTs of HI antibodies against the four influenza virus strains in the H subgroup and C subgroup ranged from 90.77 to 684.76 and from 101.25 to 913.15, respectively (**Figure 3**). The GMT ratios (C subgroup/H subgroup) ranged from 0.93 to 1.33. All lower limits of the 95% CIs exceeded the non-inferiority thresholds. For the A/H1N1 strain, the SCR in the C subgroup was higher than that in the H subgroup. For the other three strains, the SPRs, SCRs, and GMFRs were similar between the two subgroups, with all values meeting the immunogenicity criteria of European Medicines Agency for influenza vaccines. (**Table 5**).

**Table 5:** Post-vaccination antibody levels of four influenza virus strains in the separate administration population.

Subtype	Variable	H subgroup		C subgroup		Total		P value
		(N=156)		(N=140)		(N=296)		
		Value	95%CI	Value	95%CI	Value	95%CI	
A/H1N1	SPR, % (n)	140 (89.74)	83.88,94.02	134 (95.71)	90.91,98.41	274 (92.57)	88.96,95.28	0.0505
	SCR, % (n)	138 (88.46)	82.38,93.02	133 (95.00)	89.97,97.97	271 (91.55)	87.78,94.46	0.0434
	GMFR	57.53	43.70,75.72	73.52	54.75,98.72	64.60	52.87,78.94	0.2297
A/H3N2	SPR, % (n)	151 (96.79)	92.68,98.95	139 (99.29)	96.08,99.98	290 (97.97)	95.64,99.25	0.2181
	SCR, % (n)	119 (76.28)	68.82,82.72	105 (75.00)	66.98,81.93	224 (75.68)	70.38,80.45	0.7974
	GMFR	8.51	6.72,10.78	7.46	5.90,9.45	8.00	6.77,9.45	0.4381
B/Victoria	SPR, % (n)	130 (83.33)	76.54,88.81	115 (82.14)	74.78,88.10	245 (82.77)	77.98,86.89	0.7866
	SCR, % (n)	117 (75.00)	67.45,81.58	100 (71.43)	63.19,78.74	217 (73.31)	67.89,78.26	0.4880
	GMFR	7.03	5.75,8.60	7.65	6.08,9.63	7.32	6.29,8.51	0.5844
B/Yamagata	SPR, % (n)	150 (96.15)	91.82,98.58	134 (95.71)	90.91,98.41	284 (95.95)	93.03,97.89	0.8482
	SCR, % (n)	114 (73.08)	65.40,79.86	97 (69.29)	60.94,76.80	211 (71.28)	65.76,76.37	0.4717
	GMFR	5.32	4.32,6.54	5.25	4.28,6.44	5.29	4.57,6.11	0.9350

SPR, seroprotection rate (≥1:40); SCR, seroconversion rate; GMFR, geometric.

#### 3.3.4 Immunogenicity of PPSV23 in the chronic disease group and the healthy group

Before PPSV23 vaccination, the GMCs of antibodies against the 23 serotypes of Streptococcus pneumoniae in the H group and C group ranged from 0.46 to 5.14 μg/ml and from 0.39 to 4.98 μg/ml, respectively (**Table 6**). The GMCs for serotypes 3, 8, 9N, 15B, 18C, and 22F in the H group were higher than those in the C group. No differences were observed in the pre-vaccination GMCs for other serotypes between the two groups.

**Table 6:** Pre-vaccination antibodies against 23 pneumococcal serotypes.

Serotype	Variable	H group		C group		Total		*P*-value
		(N=245)		(N=204)		(N=449)		
		Value	95%CI	Value	95%CI	Value	95%CI	
1	GMC (ug/ml)	1.13	1.01,1.26	1.16	1.03,1.32	1.15	1.06-1.24	0.7382
2	GMC (ug/ml)	3.12	2.73,3.56	2.92	2.54,3.36	3.03	2.75-3.33	0.5067
3	GMC (ug/ml)	0.49	0.44,0.55	0.39	0.35,0.44	0.44	0.41-0.48	0.0088
4	GMC (ug/ml)	0.67	0.61,0.74	0.63	0.56,0.69	0.65	0.61-0.70	0.3255
5	GMC (ug/ml)	0.46	0.42,0.51	0.47	0.43,0.53	0.47	0.43-0.50	0.7123
6B	GMC (ug/ml)	1.26	1.10,1.45	1.23	1.05,1.44	1.25	1.12-1.39	0.8076
7F	GMC (ug/ml)	1.27	1.14,1.43	1.23	1.09,1.39	1.26	1.16-1.36	0.7062
8	GMC (ug/ml)	2.02	1.81,2.25	1.71	1.54,1.90	1.87	1.73-2.02	0.0364
9N	GMC (ug/ml)	2.89	2.61,3.21	2.45	2.19,2.74	2.68	2.49-2.90	0.0345
9V	GMC (ug/ml)	2.04	1.81,2.29	1.79	1.59,2.01	1.92	1.77-2.08	0.1205
10A	GMC (ug/ml)	2.21	1.98,2.47	1.98	1.75,2.24	2.10	1.94-2.28	0.1832
11A	GMC (ug/ml)	2.55	2.29,2.85	2.35	2.10,2.63	2.46	2.28-2.66	0.3007
12F	GMC (ug/ml)	0.87	0.78,0.99	0.85	0.75,0.96	0.86	0.79-0.94	0.7220
14	GMC (ug/ml)	5.14	4.55,5.81	4.98	4.41,5.62	5.07	4.65-5.52	0.7166
15B	GMC (ug/ml)	4.80	4.30,5.37	4.02	3.56,4.54	4.43	4.08-4.81	0.0335
17F	GMC (ug/ml)	1.35	1.17,1.54	1.17	1.02,1.35	1.27	1.15-1.39	0.1653
18C	GMC (ug/ml)	2.25	2.05,2.47	1.91	1.73,2.11	2.09	1.95-2.23	0.0176
19A	GMC (ug/ml)	5.11	4.69,5.57	4.86	4.45,5.31	5.00	4.70-5.31	0.4221
19F	GMC (ug/ml)	2.07	1.86,2.30	1.96	1.76,2.19	2.02	1.87-2.18	0.5025
20	GMC (ug/ml)	4.27	3.89,4.68	3.86	3.51,4.23	4.08	3.82-4.35	0.1305
22F	GMC (ug/ml)	1.77	1.59,1.96	1.50	1.35,1.67	1.64	1.53-1.77	0.0333
23F	GMC (ug/ml)	1.15	1.02,1.30	0.98	0.84,1.13	1.07	0.97-1.17	0.0913
33F	GMC (ug/ml)	3.00	2.67,3.36	2.97	2.64,3.34	2.99	2.75-3.24	0.9054

H group, Healthy group; C group, chronic disease group; GMC, geometric mean concentration.

**Figure 4:**
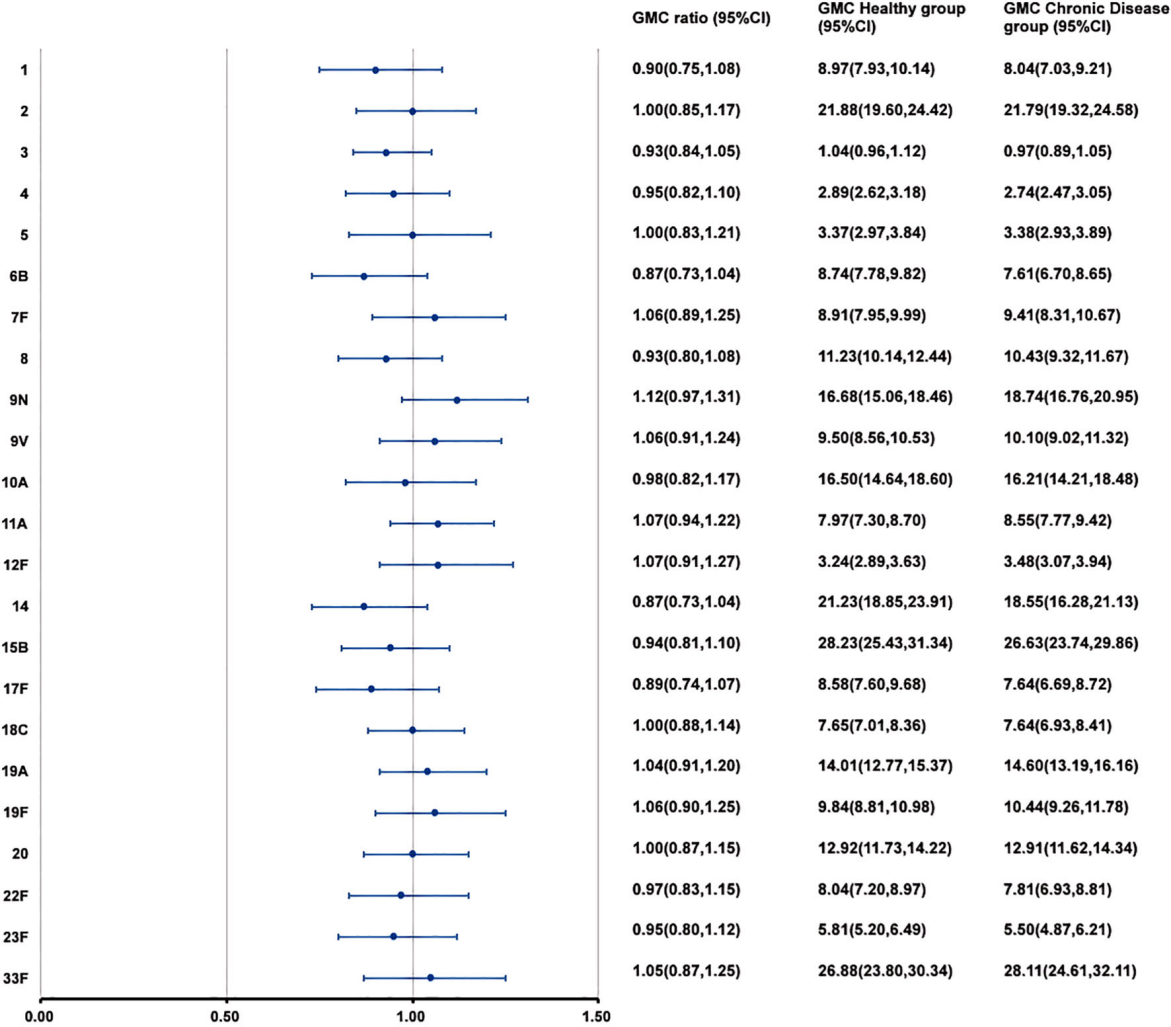
GMCs and GMC ratios of pneumococcal antibodies against 23 serotypes after vaccination. At 28 days after PPSV23 vaccination, the antibody GMCs, GMC ratios (Chronic Disease group/ Healthy group), and 95% CIs were calculated. To evaluate the non-inferiority, the lower limits of the 95% CIs for the GMC ratios needed to exceed 0.5. GMC, geometric mean concentration; PPSV, Pneumococcal polysaccharide vaccine; CI, Confidence interval.

At 28 days after PPSV23 vaccination, the adjusted GMCs of pneumococcal antibodies increased and ranged from 1.04 to 28.23 μg/mL in the H group and from 0.97 to 28.11 μg/mL in the C group(**Figure 4**). The GMC ratios (C group/H group) ranged from 0.87 to 1.12, and all the lower limits of the 95% CIs exceeded the non-inferiority thresholds. The 2-fold increase rates of pneumococcal antibodies against the 23 serotypes ranged from 44.49% to 93.47% in the H group, and from 54.41% to 93.63% in the C group (**Table 7**). For serotype 3, the 2-fold increase rates in the C group was higher than that in the H group (p=0.0363). The 2-fold increase rates for the remaining 22 serotypes were similar between the two groups. The antibody GMFRs for the 23 serotypes ranged from 2.26 to 8.99 in the H group, and from 2.27 to 9.43 in the C group. For serotype 9N, the GMFR in the C group was higher than that in the H group (p=0.0210), while for the other 22 serotypes, the GMFRs were similar between the two groups.

**Table 7:** Post-vaccination antibodies against 23 pneumococcal serotypes.

Serotype	Variable	H group		C group		Total		*P*- value
		(N=245)		(N=204)		(N=449)		
		Value	95%CI	Value	95%CI	Value	95%CI	
1	GMFR	7.85	6.90,8.94	7.00	6.14,7.99	7.45	6.80-8.18	0.2259
	2-fold increase rate, % (n)	91.43 (224)	87.20,94.62	91.18 (186)	86.41,94.69	91.31 (410)	88.32-93.75	0.9248
2	GMFR	7.16	6.37,8.04	7.30	6.37,8.35	7.22	6.61-7.88	0.8301
	2-fold increase rate, % (n)	93.47 (229)	89.61,96.22	91.67 (187)	86.99,95.07	92.65 (416)	89.83-94.89	0.4661
3	GMFR	2.26	2.07,2.47	2.27	2.08,2.47	2.27	2.13-2.41	0.9596
	2-fold increase rate, % (n)	44.49 (109)	38.16,50.95	54.41 (111)	47.31,61.38	49 (220)	44.28-53.73	0.0363
4	GMFR	4.40	3.97,4.87	4.26	3.84,4.73	4.34	4.03-4.66	0.6666
	2-fold increase rate, % (n)	83.27 (204)	77.99,87.72	84.31 (172)	78.58,89.02	83.74 (376)	80.00-87.03	0.7643
5	GMFR	7.23	6.33,8.27	7.22	6.31,8.26	7.23	6.57-7.95	0.9830
	2-fold increase rate, % (n)	89.80 (220)	85.31,93.29	90.69 (185)	85.84,94.30	90.2 (405)	87.07-92.79	0.7520
6B	GMFR	6.99	6.19,7.90	6.13	5.36,7.01	6.58	6.02-7.20	0.1525
	2-fold increase rate, % (n)	91.43 (224)	87.20,94.62	87.25 (178)	81.89,91.50	89.53 (402)	86.32-92.21	0.1503
7F	GMFR	7.07	6.31,7.92	7.53	6.59,8.60	7.27	6.67-7.93	0.4826
	2-fold increase rate, % (n)	91.84 (225)	87.67,94.94	93.63 (191)	89.35,96.56	92.65 (416)	89.83-94.89	0.4691
8	GMFR	5.81	5.19,6.51	5.78	5.13,6.53	5.80	5.34-6.29	0.9564
	2-fold increase rate, % (n)	88.16 (216)	83.44,91.93	91.67 (187)	86.99,95.07	89.76 (403)	86.57-92.40	0.2229
9N	GMFR	5.99	5.37,6.68	7.30	6.41,8.32	6.55	6.02-7.13	0.0210
	2-fold increase rate, % (n)	86.53 (212)	81.61,90.54	91.67 (187)	86.99,95.07	88.86 (399)	85.58-91.62	0.0850
9V	GMFR	4.84	4.34,5.40	5.40	4.77,6.11	5.09	4.69-5.52	0.1964
	2-fold increase rate, % (n)	86.12 (211)	81.15,90.19	85.29 (174)	79.68,89.85	85.75 (385)	82.17-88.85	0.8026
10A	GMFR	7.80	6.90,8.80	7.76	6.81,8.85	7.78	7.12-8.50	0.9636
	2-fold increase rate, % (n)	91.02 (223)	86.72,94.29	92.16 (188)	87.58,95.45	91.54 (411)	88.57-93.94	0.6666
11A	GMFR	3.19	2.90,3.51	3.53	3.18,3.93	3.34	3.12-3.59	0.1628
	2-fold increase rate, % (n)	69.80 (171)	63.63,75.48	73.53 (150)	66.92,79.45	71.49 (321)	67.07-75.63	0.3829
12F	GMFR	3.73	3.29,4.23	4.06	3.54,4.66	3.88	3.54-4.25	0.3672
	2-fold increase rate, % (n)	72.24 (177)	66.19,77.76	79.90 (163)	73.74,85.17	75.72 (340)	71.48-79.62	0.0595
14	GMFR	4.16	3.63,4.77	3.69	3.24,4.20	3.94	3.58-4.33	0.2026
	2-fold increase rate, % (n)	68.57 (168)	62.35,74.33	70.59 (144)	63.82,76.74	69.49 (312)	65.00-73.72	0.6440
15B	GMFR	6.15	5.47,6.91	6.28	5.54,7.12	6.21	5.70-6.76	0.8052
	2-fold increase rate, % (n)	87.76 (215)	82.98,91.58	88.24 (180)	83.00,92.31	87.97 (395)	84.60-90.83	0.8762
17F	GMFR	6.63	5.80,7.59	6.20	5.41,7.10	6.43	5.85-7.08	0.4872
	2-fold increase rate, % (n)	85.31 (209)	80.24,89.49	86.76 (177)	81.33,91.09	85.97 (386)	82.41-89.05	0.6577
18C	GMFR	3.57	3.24,3.93	3.78	3.43,4.16	3.66	3.42-3.92	0.4200
	2-fold increase rate, % (n)	77.14 (189)	71.37,82.25	83.33 (170)	77.50,88.17	79.96 (359)	75.95-83.56	0.1028
19A	GMFR	2.79	2.54,3.07	2.94	2.65,3.26	2.86	2.67-3.06	0.4736
	2-fold increase rate, % (n)	64.08 (157)	57.73,70.09	63.73 (130)	56.72,70.32	63.92 (287)	59.29-68.37	0.9376
19F	GMFR	4.85	4.33,5.42	5.21	4.60,5.90	5.01	4.61-5.44	0.3923
	2-fold increase rate, % (n)	82.86 (203)	77.54,87.36	85.78 (175)	80.23,90.27	84.19 (378)	80.48-87.44	0.3973
20	GMFR	3.16	2.87,3.49	3.18	2.87,3.52	3.17	2.95-3.40	0.9458
	2-fold increase rate, % (n)	66.53 (163)	60.24,72.41	69.12 (141)	62.29,75.38	67.71 (304)	63.16-72.01	0.5594
22F	GMFR	4.80	4.27,5.39	4.87	4.33,5.48	4.83	4.44-5.25	0.8632
	2-fold increase rate, % (n)	81.63 (200)	76.21,86.28	86.76 (177)	81.33,91.09	83.96 (377)	80.24-87.24	0.1400
23F	GMFR	5.32	4.75,5.95	5.31	4.63,6.08	5.31	4.87-5.80	0.9825
	2-fold increase rate, % (n)	85.31 (209)	80.24,89.49	86.27 (176)	80.78,90.68	85.75 (385)	82.17-88.85	0.7701
33F	GMFR	8.99	7.94,10.18	9.43	8.19,10.85	9.19	8.37-10.08	0.6137
	2-fold increase rate, % (n)	93.47 (229)	89.61,96.22	93.14 (190)	88.75,96.20	93.32 (419)	90.60-95.45	0.8884

H group, Healthy group; C group, Chronic Disease group; GMFR, geometric mean fold rise.

#### 3.3.5 Immunogenicity of PPSV23 in the chronic disease subgroup and the healthy subgroup after simultaneous vaccination

In the simultaneous vaccination population, the pre-vaccination pneumococcal antibody GMCs for serotypes 4, 8, and 10A in the H subgroup were higher than those in the C subgroup (p=0.0359, 0.0108, 0.0344, respectively) (**Table 8**). No differences were observed in the pre-vaccination GMCs for other serotypes between the two subgroups.

**Table 8:** Pre-vaccination antibodies against 23 pneumococcal serotypes in the simultaneous administration population.

Serotype	Variable	H subgroup		C subgroup		Total		*P*-value
		(N=91)		(N=65)		(N=156)		
		Value	95%CI	Value	95%CI	Value	95%CI	
1	GMC (ug/ml)	1.18	0.97,1.43	1.03	0.85,1.26	1.12	0.97,1.28	0.3531
2	GMC (ug/ml)	3.40	2.71,4.25	2.62	2.02,3.39	3.05	2.57,3.61	0.1330
3	GMC (ug/ml)	0.50	0.41,0.61	0.38	0.30,0.49	0.45	0.38,0.52	0.0910
4	GMC (ug/ml)	0.73	0.61,0.86	0.56	0.46,0.66	0.65	0.57,0.73	0.0359
5	GMC (ug/ml)	0.43	0.36,0.51	0.47	0.39,0.57	0.45	0.39,0.51	0.4942
6B	GMC (ug/ml)	1.16	0.94,1.44	1.22	0.89,1.67	1.19	0.99,1.42	0.7952
7F	GMC (ug/ml)	1.27	1.06,1.53	0.97	0.79,1.19	1.14	0.99,1.30	0.0526
8	GMC (ug/ml)	2.04	1.70,2.46	1.48	1.25,1.75	1.78	1.57,2.03	0.0108
9N	GMC (ug/ml)	2.83	2.42,3.30	2.34	1.94,2.83	2.61	2.32,2.94	0.1223
9V	GMC (ug/ml)	1.92	1.58,2.32	1.68	1.35,2.08	1.81	1.57,2.09	0.3617
10A	GMC (ug/ml)	2.22	1.85,2.66	1.65	1.33,2.04	1.96	1.71,2.25	0.0344
11A	GMC (ug/ml)	2.58	2.14,3.13	2.30	1.86,2.84	2.46	2.14,2.83	0.4189
12F	GMC (ug/ml)	1.14	0.96,1.36	0.99	0.81,1.23	1.08	0.94,1.23	0.3107
14	GMC (ug/ml)	4.79	4.12,5.56	4.79	3.84,5.99	4.79	4.22,5.44	0.9923
15B	GMC (ug/ml)	4.24	3.53,5.09	3.54	2.88,4.36	3.93	3.43,4.51	0.2013
17F	GMC (ug/ml)	1.21	0.98,1.50	1.13	0.87,1.47	1.18	1.00,1.39	0.6858
18C	GMC (ug/ml)	2.25	1.93,2.63	1.79	1.49,2.14	2.04	1.82,2.30	0.0564
19A	GMC (ug/ml)	5.16	4.46,5.97	4.61	3.86,5.51	4.92	4.40,5.51	0.3285
19F	GMC (ug/ml)	2.18	1.84,2.58	1.92	1.56,2.36	2.07	1.82,2.35	0.3364
20	GMC (ug/ml)	4.23	3.68,4.86	3.55	3.02,4.17	3.93	3.54,4.36	0.1045
22F	GMC (ug/ml)	1.73	1.48,2.02	1.44	1.21,1.71	1.60	1.43,1.80	0.1191
23F	GMC (ug/ml)	1.10	0.90,1.33	0.81	0.63,1.06	0.97	0.83,1.14	0.0657
33F	GMC (ug/ml)	3.42	2.88,4.07	2.84	2.24,3.60	3.17	2.75,3.65	0.1947

GMC, geometric mean concentration.

**Figure 5:**
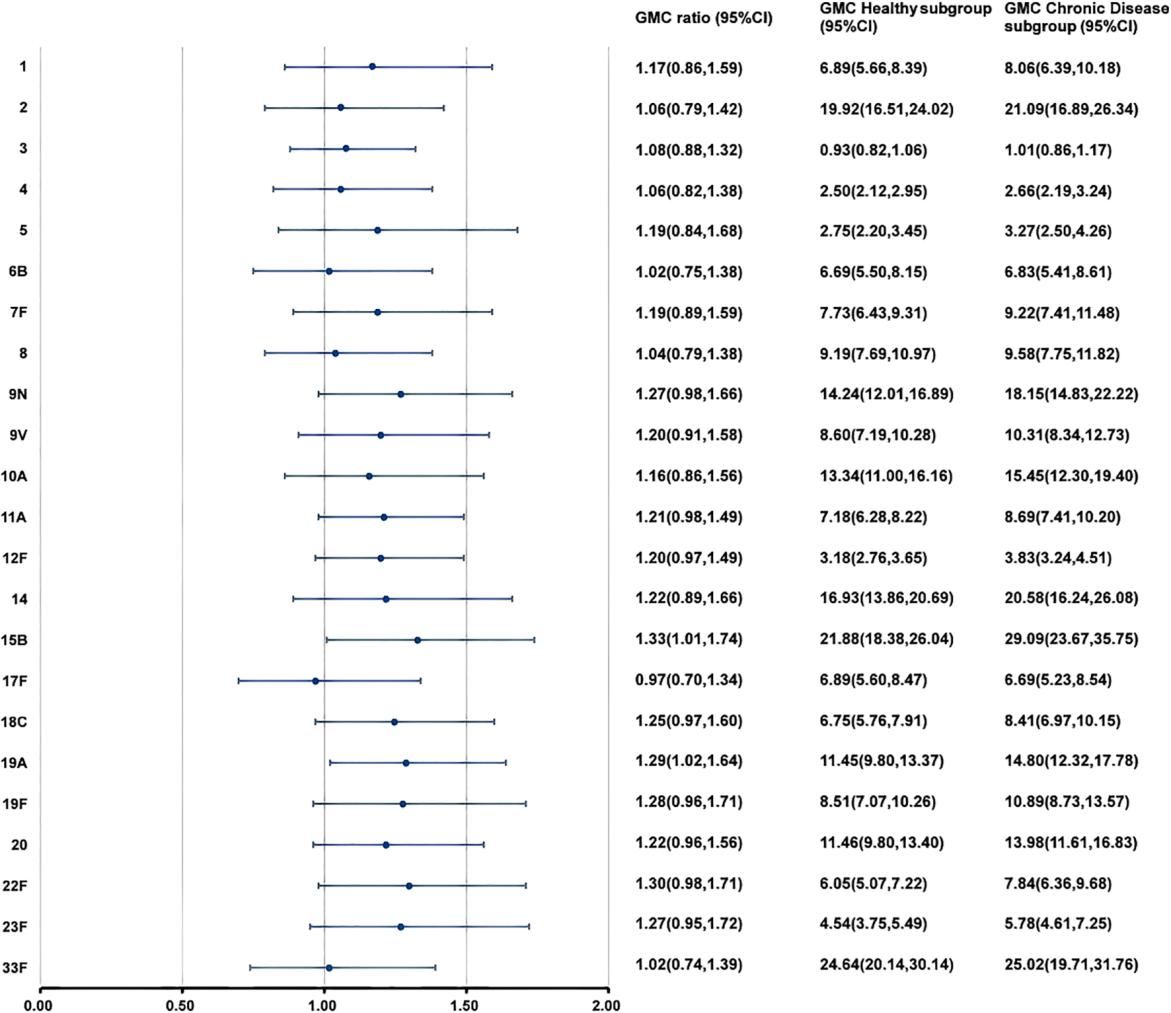
GMCs and GMC Ratios of pneumococcal antibodies in the simultaneous administration population after PPSV23 vaccination. At 28 days after PPSV23 vaccination, the antibody GMCs, GMC ratios (Chronic Disease subgroup/ Healthy subgroup), and 95% CIs were calculated. To evaluate the non-inferiority, the lower limits of the 95% CIs for the GMC ratios needed to exceed 0.5. GMC, geometric mean concentration; PPSV, Pneumococcal polysaccharide vaccine; CI, Confidence interval.

At 28 days after PPSV23 vaccination, adjusted pneumococcal antibody GMCs ranged from 0.93 to 24.64 μg/mL in the H subgroup, and from 1.01 to 29.09 μg/mL in the C subgroup (**Figure 5**). The GMC ratios (C Subgroup/H Subgroup) for the 23 pneumococcal serotypes ranged from 0.97 to 1.33, and all the lower limits of the 95% CIs exceeded the non-inferiority thresholds. The 2-fold increase rates of pneumococcal antibodies against the 23 serotypes ranged from 38.46% to 91.21% in the H subgroup, and from 50.77% to 98.46% in the C subgroup (**Table 9**). For serotypes 4, 7F, 10A, 14, 19A, and 22F, the 2-fold increases rates in the C subgroup were higher than those in the H subgroup. The 2-fold increase rates for the remaining serotypes were similar between the two subgroups. The antibody GMFRs for the 23 serotypes ranged from 2.01 to 7.62 in H subgroup, and from 2.36 to 8.43 in the C subgroup. For serotypes 9N, 12F, 15B, 18C, 19A, 22F, and 23F, the GMFRs in the C subgroup were higher than those in the H subgroup. For the other 16 serotypes, the GMFRs were similar between the two subgroups.

**Table 9:** Post-vaccination antibody levels of 23 pneumococcal serotypes in the simultaneous administration population.

Serotype	Variable	H subgroup		C subgroup		Total		*P*-value
		(N=245)		(N=204)		(N=449)		
		Value	95%CI	Value	95%CI	Value	95%CI	
1	GMFR	6.07	4.99,7.39	7.39	5.72,9.54	6.59	5.64,7.69	0.2194
	2-fold increase rate, % (n)	90.11 (82)	82.05,95.38	90.77 (59)	80.98,96.54	90.38 (141)	84.64,94.52	0.8905
2	GMFR	6.23	5.09,7.63	7.41	5.68,9.67	6.70	5.70,7.86	0.2956
	2-fold increase rate, % (n)	89.01 (81)	80.72,94.60	95.38 (62)	87.10,99.04	91.67 (143)	86.17,95.49	0.1556
3	GMFR	2.01	1.76,2.30	2.36	1.95,2.86	2.15	1.93,2.41	0.1623
	2-fold increase rate, % (n)	38.46 (35)	28.45,49.25	50.77 (33)	38.07,63.40	43.59 (68)	35.68,51.75	0.1264
4	GMFR	3.72	3.12,4.43	4.32	3.53,5.27	3.96	3.47,4.51	0.2723
	2-fold increase rate, % (n)	73.63 (67)	63.35,82.31	87.69 (57)	77.18,94.53	79.49 (124)	72.29,85.53	0.0320
5	GMFR	6.19	4.89,7.82	7.25	5.61,9.37	6.61	5.57,7.85	0.3706
	2-fold increase rate, % (n)	87.91 (80)	79.40,93.81	87.69 (57)	77.18,94.53	87.82 (137)	81.64,92.51	0.9670
6B	GMFR	5.67	4.67,6.89	5.72	4.43,7.39	5.69	4.88,6.64	0.9533
	2-fold increase rate, % (n)	87.91 (80)	79.40,93.81	87.69 (57)	77.18,94.53	87.82 (137)	81.64,92.51	0.9670
7F	GMFR	6.63	5.54,7.92	8.43	6.62,10.73	7.32	6.34,8.46	0.1052
	2-fold increase rate, % (n)	90.11 (82)	82.05,95.38	98.46 (64)	91.72,99.96	93.59 (146)	88.53,96.88	0.0463
8	GMFR	4.90	4.13,5.81	5.75	4.49,7.37	5.24	4.54,6.04	0.2727
	2-fold increase rate, % (n)	84.62 (77)	75.54,91.33	89.23 (58)	79.06,95.56	86.54 (135)	80.16,91.47	0.4050
9N	GMFR	5.26	4.41,6.27	7.30	5.78,9.20	6.03	5.23,6.95	0.0243
	2-fold increase rate, % (n)	83.52 (76)	74.27,90.47	93.85 (61)	84.99,98.30	87.82 (137)	81.64,92.51	0.0518
9V	GMFR	4.65	3.85,5.61	5.85	4.64,7.38	5.12	4.42,5.92	0.1257
	2-fold increase rate, % (n)	83.52 (76)	74.27,90.47	89.23 (58)	79.06,95.56	85.90 (134)	79.43,90.95	0.3120
10A	GMFR	6.72	5.47,8.26	8.01	6.54,9.82	7.23	6.25,8.37	0.2420
	2-fold increase rate, % (n)	87.91 (80)	79.40,93.81	96.92 (63)	89.32,99.63	91.67 (143)	86.17,95.49	0.0447
11A	GMFR	2.86	2.48,3.31	3.63	2.97,4.44	3.16	2.81,3.56	0.0507
	2-fold increase rate, % (n)	67.03 (61)	56.39,76.53	72.31 (47)	59.81,82.69	69.23 (108)	61.35,76.36	0.4816
12F	GMFR	2.87	2.48,3.33	3.67	2.99,4.49	3.18	2.82,3.59	0.0485
	2-fold increase rate, % (n)	68.13 (62)	57.53,77.51	73.85 (48)	61.46,83.97	70.51 (110)	62.69,77.53	0.4403
14	GMFR	3.54	2.84,4.40	4.29	3.39,5.44	3.83	3.27,4.50	0.2371
	2-fold increase rate, % (n)	59.34 (54)	48.53,69.52	76.92 (50)	64.81,86.47	66.67 (104)	58.68,74.00	0.0216
15B	GMFR	5.39	4.50,6.45	7.73	6.07,9.86	6.27	5.41,7.25	0.0159
	2-fold increase rate, % (n)	85.71 (78)	76.81,92.17	90.77 (59)	80.98,96.54	87.82 (137)	81.64,92.51	0.3412
17F	GMFR	5.80	4.62,7.28	5.74	4.53,7.28	5.78	4.90,6.80	0.9505
	2-fold increase rate, % (n)	84.62 (77)	75.54,91.33	84.62 (55)	73.52,92.37	84.62 (132)	77.98,89.89	1.0000
18C	GMFR	3.22	2.75,3.77	4.28	3.49,5.24	3.62	3.19,4.11	0.0272
	2-fold increase rate, % (n)	71.43 (65)	61.00,80.41	83.08 (54)	71.73,91.24	76.28 (119)	68.82,82.72	0.0917
19A	GMFR	2.30	1.96,2.70	3.05	2.53,3.67	2.59	2.29,2.92	0.0243
	2-fold increase rate, % (n)	45.05 (41)	34.60,55.84	67.69 (44)	54.95,78.77	54.49 (85)	46.33,62.47	0.0051
19F	GMFR	4.03	3.32,4.90	5.42	4.26,6.90	4.56	3.92,5.31	0.0568
	2-fold increase rate, % (n)	76.92 (70)	66.91,85.11	87.69 (57)	77.18,94.53	81.41 (127)	74.41,87.18	0.0883
20	GMFR	2.96	2.51,3.49	3.49	2.93,4.17	3.17	2.81,3.58	0.1805
	2-fold increase rate, % (n)	60.44 (55)	49.64,70.54	75.38 (49)	63.13,85.23	66.67 (104)	58.68,74.00	0.0509
22F	GMFR	3.69	3.10,4.38	5.05	3.99,6.38	4.20	3.65,4.84	0.0286
	2-fold increase rate, % (n)	70.33 (64)	59.84,79.45	84.62 (55)	73.52,92.37	76.28 (119)	68.82,82.72	0.0386
23F	GMFR	4.45	3.73,5.31	6.39	4.77,8.57	5.18	4.41,6.07	0.0365
	2-fold increase rate, % (n)	82.42 (75)	73.02,89.60	90.77 (59)	80.98,96.54	85.90 (134)	79.43,90.95	0.1395
33F	GMFR	7.62	6.23,9.31	8.14	6.29,10.55	7.83	6.69,9.17	0.6801
	2-fold increase rate, % (n)	91.21 (83)	83.41,96.13	92.31 (60)	82.95,97.46	91.67 (143)	86.17,95.49	0.8066

GMFR, geometric mean fold rise.

#### 3.3.6 Immunogenicity of PPSV23 in the chronic disease subgroup and the healthy subgroup after separate vaccination

In the separate vaccination population, the pre-vaccination pneumococcal antibody GMC for serotype 3 in the H subgroup was higher than that in the C subgroup (p=0.0447) (**Table 10**). No differences were observed in the pre-vaccination GMCs for other serotypes between the two subgroups.

**Table 10:** Pre-vaccination antibody levels of 23 pneumococcal serotype in the separate administration population.

Serotype	Variable	H subgroup		C subgroup		Total		P value
		(N=154 )		(N=139 )		(N=293 )		
		Value	95%CI	Value	95%CI	Value	95%CI	
1	GMC (ug/ml)	1.10	0.96,1.26	1.23	1.05,1.44	1.16	1.05,1.29	0.3044
2	GMC (ug/ml)	2.96	2.51,3.49	3.07	2.61,3.63	3.01	2.68,3.39	0.7513
3	GMC (ug/ml)	0.49	0.42,0.56	0.40	0.35,0.45	0.44	0.40,0.49	0.0447
4	GMC (ug/ml)	0.64	0.57,0.73	0.66	0.58,0.75	0.65	0.60,0.71	0.7487
5	GMC (ug/ml)	0.48	0.42,0.55	0.48	0.42,0.54	0.48	0.44,0.52	0.9261
6B	GMC (ug/ml)	1.32	1.10,1.59	1.23	1.02,1.49	1.28	1.12,1.46	0.5934
7F	GMC (ug/ml)	1.27	1.10,1.48	1.38	1.19,1.60	1.32	1.19,1.47	0.4447
8	GMC (ug/ml)	2.00	1.74,2.30	1.84	1.61,2.10	1.92	1.75,2.11	0.3877
9N	GMC (ug/ml)	2.93	2.55,3.37	2.51	2.18,2.88	2.72	2.47,3.00	0.1205
9V	GMC (ug/ml)	2.11	1.83,2.44	1.84	1.61,2.12	1.98	1.79,2.19	0.1848
10A	GMC (ug/ml)	2.21	1.92,2.55	2.16	1.85,2.51	2.18	1.97,2.42	0.8158
11A	GMC (ug/ml)	2.54	2.22,2.90	2.38	2.08,2.71	2.46	2.24,2.70	0.4970
12F	GMC (ug/ml)	0.75	0.64,0.87	0.79	0.68,0.92	0.76	0.69,0.85	0.6358
14	GMC (ug/ml)	5.36	4.51,6.38	5.07	4.38,5.86	5.22	4.66,5.85	0.6233
15B	GMC (ug/ml)	5.17	4.50,5.95	4.26	3.67,4.96	4.72	4.26,5.23	0.0642
17F	GMC (ug/ml)	1.43	1.20,1.71	1.19	1.01,1.41	1.31	1.16,1.48	0.1366
18C	GMC (ug/ml)	2.25	2.00,2.53	1.97	1.75,2.22	2.11	1.94,2.30	0.1163
19A	GMC (ug/ml)	5.08	4.57,5.65	4.98	4.51,5.51	5.04	4.68,5.41	0.7907
19F	GMC (ug/ml)	2.00	1.74,2.30	1.98	1.74,2.25	1.99	1.81,2.19	0.9177
20	GMC (ug/ml)	4.29	3.80,4.85	4.01	3.57,4.50	4.16	3.82,4.52	0.4259
22F	GMC (ug/ml)	1.79	1.56,2.06	1.53	1.35,1.75	1.66	1.51,1.83	0.1185
23F	GMC (ug/ml)	1.18	1.00,1.38	1.06	0.89,1.26	1.12	1.00,1.26	0.3821
33F	GMC (ug/ml)	2.77	2.38,3.23	3.03	2.65,3.47	2.89	2.62,3.20	0.3806

GMC, geometric mean concentration.

**Figure 6:**
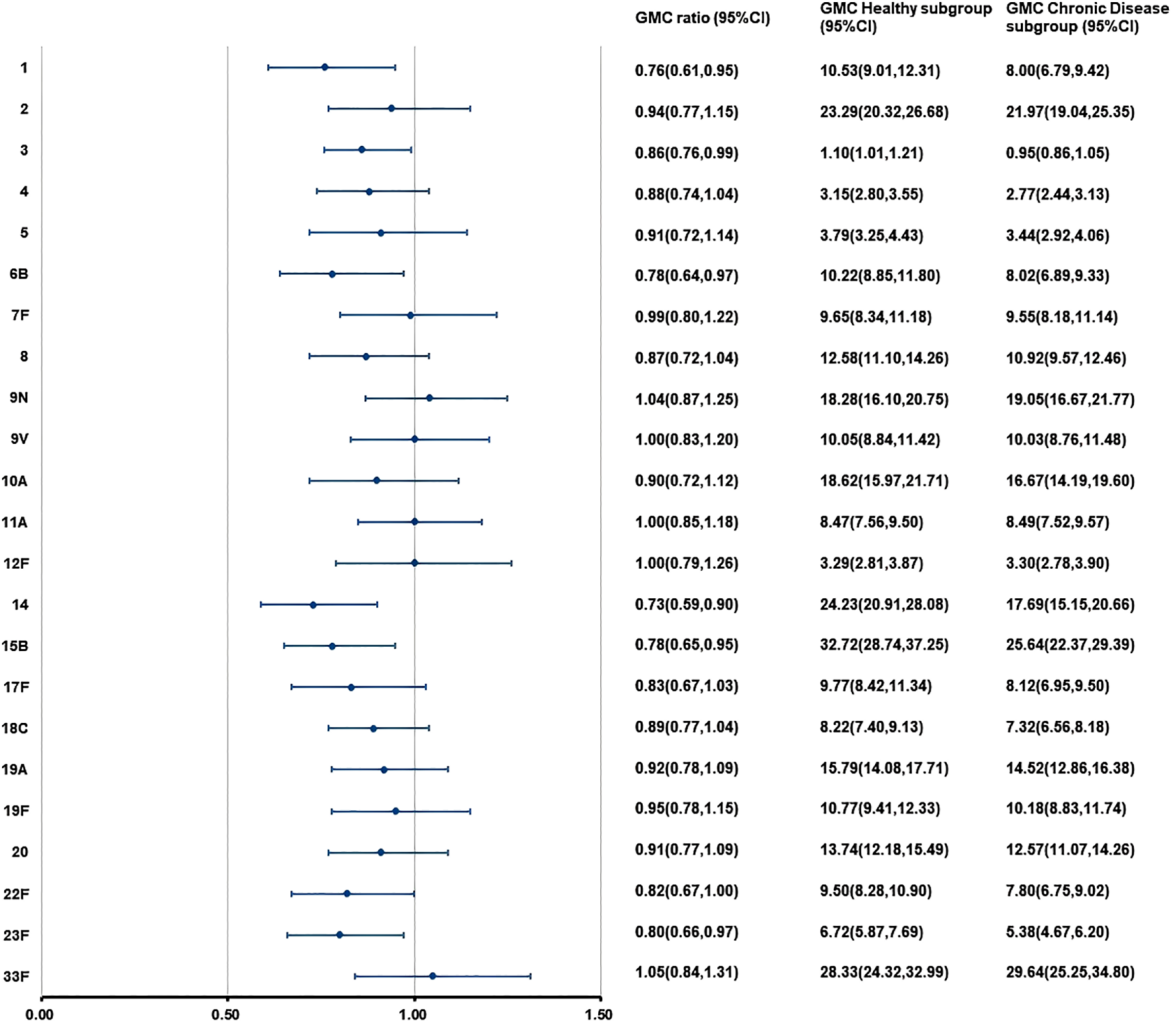
GMCs and GMC Ratios of pneumococcal antibodies in the separate administration population after PPSV23 vaccination. At 28 days after PPSV23 vaccination, the antibody GMCs, GMC ratios (Chronic Disease subgroup/ Healthy subgroup), and 95% CIs were calculated. To evaluate the non-inferiority, the lower limits of the 95% CIs for the GMC ratios needed to exceed 0.5. GMC, geometric mean concentration; PPSV, Pneumococcal polysaccharide vaccine; CI, Confidence interval.

At 28 days after PPSV23 vaccination, adjusted pneumococcal antibody GMCs ranged from 1.10 to 32.72 μg/mL in the H subgroup, and from 0.95 to 29.64 μg/mL in the C subgroup (**Figure 6**). The GMC ratios (C subgroup/H subgroup) for the 23 pneumococcal serotypes ranged from 0.73 to 1.05, and all the lower limits of the 95% CIs exceeded the non-inferiority thresholds. The 2-fold increase rates of pneumococcal antibodies against the 23 serotypes ranged from 48.05% to 96.10% in the H subgroup, and from 56.12% to 93.53% in the C subgroup (**Table 11**). For serotypes 2, 19A, the 2-fold increase rates in the H subgroup were higher than those in the C subgroup. The 2-fold increase rates for the remaining serotypes were similar between the two subgroups. The antibody GMFRs for the 23 serotypes ranged from 2.42 to 9.91 in H subgroup, and from 2.23 to 10.10 in the C subgroup, For serotypes 1, 6B, and 14, the GMFRs in the H subgroup were higher than those in the C subgroup. For the other 20 serotypes, the GMFRs were similar between the two subgroups.

**Table 11:** Post-vaccination antibody levels of 23 pneumococcal serotypes in the separate administration population.

Serotype	Variable	H subgroup		C subgroup		Total		P value
		(N=154)		(N=139)		(N=293)		
		Value	95%CI	Value	95%CI	Value	95%CI	
1	GMFR	9.14	7.73,10.80	6.83	5.85,7.97	7.96	7.10,8.93	0.0125
	2-fold increase rate, % (n)	92.21 (142)	86.78,95.91	91.37 (127)	85.41,95.46	91.81 (269)	88.06,94.68	0.7932
2	GMFR	7.77	6.74,8.94	7.24	6.19,8.47	7.51	6.77,8.34	0.5133
	2-fold increase rate, % (n)	96.10 (148)	91.71,98.56	89.93 (125)	83.68,94.38	93.17 (273)	89.65,95.78	0.0363
3	GMFR	2.42	2.17,2.71	2.23	2.03,2.44	2.33	2.16,2.50	0.2522
	2-fold increase rate, % (n)	48.05 (74)	39.94,56.24	56.12 (78)	47.45,64.51	51.88 (152)	45.99,57.72	0.1678
4	GMFR	4.86	4.29,5.50	4.23	3.74,4.79	4.55	4.17,4.97	0.1252
	2-fold increase rate, % (n)	88.96 (137)	82.91,93.44	82.73 (115)	75.41,88.61	86.01 (252)	81.50,89.77	0.1250
5	GMFR	7.93	6.75,9.32	7.20	6.14,8.45	7.58	6.77,8.49	0.4027
	2-fold increase rate, % (n)	90.91 (140)	85.22,94.94	92.09 (128)	86.28,95.98	91.47 (268)	87.66,94.40	0.7187
6B	GMFR	7.91	6.78,9.23	6.33	5.40,7.41	7.12	6.37,7.95	0.0467
	2-fold increase rate, % (n)	93.51 (144)	88.38,96.84	87.05 (121)	80.31,92.14	90.44 (265)	86.48,93.56	0.0605
7F	GMFR	7.35	6.34,8.53	7.14	6.08,8.38	7.25	6.50,8.08	0.7908
	2-fold increase rate, % (n)	92.86 (143)	87.58,96.38	91.37 (127)	85.41,95.46	92.15 (270)	88.45,94.96	0.6358
8	GMFR	6.43	5.54,7.45	5.80	5.06,6.64	6.12	5.54,6.77	0.3152
	2-fold increase rate, % (n)	90.26 (139)	84.44,94.45	92.81 (129)	87.17,96.50	91.47 (268)	87.66,94.40	0.4360
9N	GMFR	6.46	5.62,7.43	7.30	6.23,8.56	6.85	6.17,7.60	0.2526
	2-fold increase rate, % (n)	88.31 (136)	82.16,92.92	90.65 (126)	84.54,94.93	89.42 (262)	85.32,92.70	0.5163
9V	GMFR	4.96	4.34,5.68	5.20	4.48,6.02	5.07	4.59,5.60	0.6473
	2-fold increase rate, % (n)	87.66 (135)	81.41,92.41	83.45 (116)	76.21,89.21	85.67 (251)	81.12,89.47	0.3046
10A	GMFR	8.51	7.32,9.89	7.65	6.46,9.06	8.09	7.23,9.05	0.3495
	2-fold increase rate, % (n)	92.86 (143)	87.58,96.38	89.93 (125)	83.68,94.38	91.47 (268)	87.66,94.40	0.3702
11A	GMFR	3.41	3.00,3.87	3.49	3.08,3.96	3.45	3.15,3.77	0.7908
	2-fold increase rate, % (n)	71.43 (110)	63.60,78.41	74.10 (103)	65.99,81.15	72.70 (213)	67.21,77.72	0.6082
12F	GMFR	4.35	3.65,5.19	4.26	3.56,5.09	4.31	3.80,4.88	0.8649
	2-fold increase rate, % (n)	74.68 (115)	67.05,81.33	82.73 (115)	75.41,88.61	78.50 (230)	73.35,83.06	0.0936
14	GMFR	4.59	3.85,5.46	3.43	2.94,4.01	4.00	3.55,4.50	0.0150
	2-fold increase rate, % (n)	74.03 (114)	66.35,80.75	67.63 (94)	59.17,75.31	70.99 (208)	65.43,76.12	0.2280
15B	GMFR	6.64	5.70,7.73	5.70	4.93,6.59	6.17	5.56,6.86	0.1522
	2-fold increase rate, % (n)	88.96 (137)	82.91,93.44	87.05 (121)	80.31,92.14	88.05 (258)	83.78,91.54	0.6146
17F	GMFR	7.18	6.08,8.48	6.42	5.43,7.60	6.81	6.05,7.66	0.3541
	2-fold increase rate, % (n)	85.71 (132)	79.17,90.83	87.77 (122)	81.14,92.71	86.69 (254)	82.26,90.36	0.6050
18C	GMFR	3.79	3.35,4.30	3.56	3.20,3.97	3.68	3.39,4.00	0.4551
	2-fold increase rate, % (n)	80.52 (124)	73.37,86.45	83.45 (116)	76.21,89.21	81.91 (240)	77.02,86.15	0.5148
19A	GMFR	3.13	2.80,3.50	2.89	2.55,3.27	3.01	2.77,3.28	0.3444
	2-fold increase rate, % (n)	75.32 (116)	67.74,81.91	61.87 (86)	53.25,69.97	68.94 (202)	63.30,74.20	0.0129
19F	GMFR	5.40	4.72,6.19	5.12	4.42,5.92	5.27	4.77,5.81	0.5899
	2-fold increase rate, % (n)	86.36 (133)	79.91,91.36	84.89 (118)	77.84,90.40	85.67 (251)	81.12,89.47	0.7196
20	GMFR	3.29	2.91,3.72	3.04	2.68,3.45	3.17	2.90,3.46	0.3801
	2-fold increase rate, % (n)	70.13 (108)	62.24,77.23	66.19 (92)	57.68,73.99	68.26 (200)	62.59,73.55	0.4691
22F	GMFR	5.61	4.82,6.53	4.79	4.17,5.49	5.20	4.69,5.77	0.1303
	2-fold increase rate, % (n)	88.31 (136)	82.16,92.92	87.77 (122)	81.14,92.71	88.05 (258)	83.78,91.54	0.8864
23F	GMFR	5.91	5.11,6.83	4.87	4.20,5.64	5.39	4.86,5.98	0.0642
	2-fold increase rate, % (n)	87.01 (134)	80.66,91.88	84.17 (117)	77.02,89.81	85.67 (251)	81.12,89.47	0.4884
33F	GMFR	9.91	8.47,11.60	10.10	8.54,11.95	10.00	8.92,11.21	0.8734
	2-fold increase rate, % (n)	94.81 (146)	90.02,97.73	93.53 (130)	88.06,97.00	94.20 (276)	90.87,96.58	0.6398

GMFR, geometric mean fold rise.

### 3.4 Safety

Within 28 days after vaccination with IIV4 and PPSV23, the rate and severity of any type of adverse events were comparable between the H group and the C Disease group (**Table 12**). The overall rates of adverse events in the two groups were 1.47% (4/272) in the Healthy group and 0.96% (2/208) in the Chronic Disease group, with most events being mild (grade 1). The main symptoms in the two groups were both the pain at the injection site. In the Healthy group, one participant reported grade 2 adverse events (fever and weakness) and a grade 1 cough. No grade 2 adverse events or systemic adverse events were observed in the Chronic Disease group.

**Table 12:** The rates and severity of adverse events.

Adverse Events	H group (N=272)	C group (N=208)	Total (N=480)	*P*-value
Overall	4(1.47)	2(0.96)	6(1.25)	0.7021
Grade 1	4(1.47)	2(0.96)	6(1.25)	0.7021
Grade 2	1(0.37)	0	1(0.21)	1.0000
Local	3(1.10)	2(0.96)	5(1.04)	1.0000
Grade 1	3(1.10)	2(0.96)	5(1.04)	1.0000
Pain	3(1.10)	2(0.96)	5(1.04)	1.0000
Grade 1	3(1.10)	2(0.96)	5(1.04)	1.0000
Induration/ Swelling	0	1(0.48)	1(0.21)	0.4333
Grade 1	0	1(0.48)	1(0.21)	0.4333
Redness	1(0.37)	1(0.48)	2(0.42)	1.0000
Grade 1	1(0.37)	1(0.48)	2(0.42)	1.0000
Systemic	1(0.37)	0	1(0.21)	1.0000
Grade 1	1(0.37)	0	1(0.21)	1.0000
Grade 2	1(0.37)	0	1(0.21)	1.0000
Cough	1(0.37)	0	1(0.21)	1.0000
Grade 1	1(0.37)	0	1(0.21)	1.0000
Fever	1(0.37)	0	1(0.21)	1.0000
Grade 2	1(0.37)	0	1(0.21)	1.0000
Fatigue	1(0.37)	0	1(0.21)	1.0000
Grade 2	1(0.37)	0	1(0.21)	1.0000

## 4 Discussion

Our study evaluated an elderly population aged 60 years and above with chronic diseases, most of whom suffered from chronic conditions such as hypertension, obesity, and diabetes. These comorbidities render them more susceptible to complications arising from influenza and pneumococcal infections. The results demonstrated that, at 28 days after vaccination with inactivated quadrivalent influenza vaccine (IIV4) and 23-valent pneumococcal polysaccharide vaccine (PPSV23), the post-vaccination influenza antibody geometric mean titers (GMTs) against the four influenza virus strains and pneumococcal antibody geometric mean concentrations (GMCs) against the 23 pneumococcal serotypes in the chronic disease group were all non-inferior to those in the healthy group. Previous studies have shown that co-administration of IIV4 and PPV23 does not reduce antibody responses that reflect protective efficacy against influenza or pneumococcal diseases (26, 27). Our study further supports the application value of this co-administration strategy in elderly individuals with chronic conditions.

Currently, the determination of the margin for non-inferiority trials requires a comprehensive consideration of multiple factors, including vaccine type, disease characteristics, practical clinical needs, and national regulatory specifications, which is established through systematic validation (28). In conventional practice, the lower limit of the 95% confidence interval (CI) for the ratio of geometric mean titer (GMT) or geometric mean concentration (GMC) (test/reference) is generally set at 0.67. The World Health Organization (WHO) has indicated that under certain circumstances, national regulatory authorities (NRAs) may consider allowing a lower bound (e.g., 0.5) or alternative criteria for determining vaccine non-inferiority (29). In accordance with the regulatory requirements specified by the National Medical Products Administration (NMPA) of China, a non-inferiority margin of 0.5 is acceptable for multivalent vaccines (21). Furthermore, referencing other clinical research, particularly those for pneumococcal vaccine studies, a margin of 0.5 is commonly adopted (27, 30). Based on the aforementioned considerations, a non-inferiority margin of 0.5 was selected for the present study.

Additionally, the SPRs, SCRs, and GMRFs of influenza antibodies against four strains in the Chronic Disease group were all similar to those in the Healthy group. And the immunogenicity profiles of IIV4 in the two groups all met the EMA evaluation criteria. The 2-fold increase rates and GMRFs of pneumonia antibodies against 23 serotypes in the Chronic Disease group were all similar to or higher than those in the Healthy group. Similarly, for the simultaneous administration or separate administration, the immunogenicity profiles in the Chronic Disease subgroup were also comparable to those in the Healthy subgroup. Moreover, the rates of adverse events in the Chronic Disease group after vaccination with the two vaccines were low. These findings demonstrated that administration, especially simultaneous vaccination, of IIV4 and PPSV23 in individuals with chronic disease exhibited favorable immunogenicity and safety profiles. This study provided the first clinical evidence on immunogenicity and safety of simultaneous vaccination with IIV4 and PPSV23 in individuals with chronic diseases.

Simultaneous vaccination could provide more comprehensive vaccination protection, reduce missed opportunities for vaccination (MOV), and improve the coverage and timeliness of vaccination (31–33). However, clinical evidence on simultaneous vaccination with these two vaccines in individuals with chronic diseases is still lacking. Given that the safety and immunogenicity risks in individuals with chronic diseases may be higher than healthy individuals, we conducted the clinical trials sequentially. In our previous study, we conducted a clinical trial to evaluate the safety of simultaneous vaccination in this population (34). The results were consistent with those of this study, showing that the safety profiles of simultaneous vaccination in chronic disease population were comparable to those in healthy populations. Based on these findings, we conducted this clinical trial to further evaluate the immunogenicity of simultaneous vaccination in chronic disease population. And the results of this clinical trial demonstrated the comparable immunogenicity and safety profiles of simultaneous vaccination between individuals with chronic diseases and healthy individuals. In addition, we conducted an additional 3-year safety follow-up through China’s Adverse Event Following Immunization (AEFI) reporting system, and no new adverse events were reported by the participants, supporting the simultaneous vaccination strategy in this populations. Relevant studies have shown that simultaneous vaccination can cause an increase in C-reactive protein (35). However, an elevation in C-reactive protein does not necessarily lead to specific clinical manifestations. In this study, only adverse events with clinical symptoms were collected, and adverse events related to laboratory indicators were not evaluated. The safety assessment of laboratory indicators will be carried out in subsequent post-marketing clinical trials.

Hypertension is a common chronic disease, particularly among the older adults (36). It is estimated that 1.28 billion adults aged 30 to 79 years worldwide suffer from hypertension (37). In 2018, the weighted prevalence of hypertension among adults aged 18 years and older in China was 27.5% (38). To date, clinical evidence on the immunogenicity of IIV4 and PPSV23 vaccination in hypertensive populations remains limited. In the chronic disease group in this clinical trials, individuals with hypertension accounted for 63.41%. The results showed that the post-vaccination GMTs of HI antibodies against four influenza virus strains and GMCs of 23-valent pneumococcal antibodies in the hypertensive group were all non-inferior to those in the healthy group (**Supplementary Figures S1**, **S2**). The post-vaccination SPRs, SCRs, and GMRFs of influenza antibodies and the 2-fold increase rates and GMRFs of pneumonia antibodies in the hypertensive group were all similar to those in the healthy group (**Supplementary Tables S2**, **S3**). These findings suggested that antibody levels in hypertensive individuals following IIV4 and PPSV23 vaccination were comparable to those in healthy individuals.

However, this clinical trial still had some limitations. First, it evaluated the short-term immunogenicity and safety of two vaccines. Long-term follow-up studies would be needed to evaluate long-term safety and immune persistence. Second, the sample size was limited, and the study may not fully observe all low-incidence adverse events. In addition, further clinical trials are needed to analyzed and validated the immunogenicity and safety profiles of simultaneous vaccination in broader population. Specially, age-stratified (children and young adults) and disease-stratified (e.g., diabetes mellitus, chronic obstructive pulmonary disease, asthma, chronic cardiovascular disorder and compromised immunity) randomized controlled clinical trials that compare immunogenicity and safety profiles of simultaneous vaccination in individuals with chronic diseases versus healthy individuals would be helpful to establish optimal vaccination strategies for these populations.

## 5 Conclusions

In conclusion, the immunogenicity and safety profiles of IIV4 and PPSV23 vaccination, especially simultaneous vaccination, in individuals with chronic diseases were comparable to those in healthy individuals. This study supports the vaccination strategy, particularly simultaneous vaccination, of two vaccines in chronic disease populations.

## Data Availability

The original contributions presented in the study are included in the article/**Supplementary Material**. Further inquiries can be directed to the corresponding authors.
