# Correction: The immunogenicity and safety of an inactivated quadrivalent influenza vaccine and a 23-valent pneumococcal polysaccharide vaccine in individuals with chronic diseases

**DOI:** 10.3389/fimmu.2025.1682368

**Published:** 2025-08-20

**Authors:** Frontiers Production Office

**Affiliations:** Frontiers Media SA, Lausanne, Switzerland

**Keywords:** chronic disease, inactivated quadrivalent influenza vaccine, 23-valent pneumococcal polysaccharide vaccine, simultaneous administration, immunogenicity, safety

The Reviewer Ahmed Tawfik Moustafa’s affiliation “Institut Pasteur de Paris, France” was erroneously given as “Institut Pasteur de Lille, France”.

The original version of this article has been updated.

