# Supplementary material for: The immunogenicity and safety of an inactivated quadrivalent influenza vaccine and a 23-valent pneumococcal polysaccharide vaccine in individuals with chronic diseases
Source: Front Immunol. 2025 Aug 1;16:1624095. doi: 10.3389/fimmu.2025.1624095 (PMC12355927; doi:10.3389/fimmu.2025.1624095)
Supplement: Supplementary file 1 [file DataSheet1.docx]

Supplementary Material

# Contents:

1. Inclusion and exclusion criteria

2. Tables and figures

Table S1. List of Pre-existing Chronic Diseases.

Figure S1. Post-vaccination GMTs and GMT ratios for HI antibodies against four influenza virus strains (Hypertension group).

Table S2. Post-vaccination antibody levels of four influenza virus strains ( Hypertension group ).

Figure S2. Post-vaccination GMCs and GMC ratios for pneumococcal antibodies of 23 serotypes (Hypertension group).

Table S3. Post-vaccination antibody levels of 23 pneumococcal serotypes ( Hypertension group ).

1. Inclusion and exclusion criteria

1.1 Inclusion criteria

Participant who:

(1) was 60 years of age or older;

(2) could understand and voluntarily sign the informed consent form;

(3) could provide legal identification.

1.2 Exclusion criteria

Participant who:

(1) had received the 2021-2022 seasonal influenza vaccine prior to the screening;

(2) had received PPSV23 within the past five years;

(3) had a documented history of severe allergic reactions to the two vaccines;

(4) had been diagnosed with uncontrolled epilepsy or other severe neurological condition (such as transverse myelitis, Guillain-Barre syndrome and demyelinating disease);

(5) were currently suffering from fever, acute exacerbation of a chronic condition, uncontrolled serious chronic disease or acute illness;

(6) had received other investigational drugs within the past 30 days;

(7) had been received live attenuated vaccines within the past 14 days;

(8) had been received any subunit or inactivated vaccines within the past 7 days;

(9)had any additional risk factors that the investigator considered unsuitable for participation.

**2. Tables and figures**

Table S1 List of Pre-existing Chronic Diseases

| **System classification and disease** | **Simultaneous administration population** |  | **Separate administration population** | | |  | **Total** |  |
| --- | --- | --- | --- | --- | --- | --- | --- | --- |
|  |  |  | **1** | **2** | **Pooled** |  |  |  |
|  | **(N=160)** |  | **(N=160)** | **(N=160)** | **(N=320)** |  | **(N=480)** |  |
| All diseases | 65(40.63) |  | 77(48.13) | 66(41.25) | 143(44.69) |  | 208(43.33) |  |
| Vascular and lymphatic diseases | 47(29.38) |  | 45(28.13) | 39(24.38) | 84(26.25) |  | 131(27.29) |  |
| Hypertension | 47(29.38) |  | 45(28.13) | 39(24.38) | 84(26.25) |  | 131(27.29) |  |
| Metabolism and nutrition disorders | 19(11.88) |  | 28(17.50) | 25(15.63) | 53(16.56) |  | 72(15.00) |  |
| Obesity | 12(7.50) |  | 14(8.75) | 12(7.50) | 26(8.13) |  | 38(7.92) |  |
| Diabetes | 6(3.75) |  | 10(6.25) | 8(5.00) | 18(5.63) |  | 24(5.00) |  |
| Hyperglycemia | 1(0.63) |  | 0(0.00) | 0(0.00) | 0(0.00) |  | 1(0.21) |  |
| Hyperlipidemia | 1(0.63) |  | 5(3.13) | 5(3.13) | 10(3.13) |  | 11(2.29) |  |
| Gout | 0(0.00) |  | 1(0.63) | 0(0.00) | 1(0.31) |  | 1(0.21) |  |
| Ear and labyrinth diseases | 1(0.63) |  | 1(0.63) | 0(0.00) | 1(0.31) |  | 2(0.42) |  |
| Dizziness | 1(0.63) |  | 1(0.63) | 0(0.00) | 1(0.31) |  | 2(0.42) |  |
| Diseases of liver and biliary system | 1(0.63) |  | 2(1.25) | 0(0.00) | 2(0.63) |  | 3(0.63) |  |
| Cholelithiasis | 1(0.63) |  | 1(0.63) | 0(0.00) | 1(0.31) |  | 2(0.42) |  |
| Hepatic cirrhosis | 0(0.00) |  | 1(0.63) | 0(0.00) | 1(0.31) |  | 1(0.21) |  |
| Infections and infestations | 0(0.00) |  | 0(0.00) | 3(1.88) | 3(0.94) |  | 3(0.63) |  |
| Viral hepatitis | 0(0.00) |  | 0(0.00) | 1(0.63) | 1(0.31) |  | 1(0.21) |  |
| Pulmonary tuberculosis | 0(0.00) |  | 0(0.00) | 2(1.25) | 2(0.63) |  | 2(0.42) |  |
| Investigations | 3(1.88) |  | 1(0.63) | 3(1.88) | 4(1.25) |  | 7(1.46) |  |
| High uric acid | 0(0.00) |  | 0(0.00) | 1(0.63) | 1(0.31) |  | 1(0.21) |  |
| Heart rate decreased | 1(0.63) |  | 0(0.00) | 0(0.00) | 0(0.00) |  | 1(0.21) |  |
| Heart rate increased | 0(0.00) |  | 0(0.00) | 1(0.63) | 1(0.31) |  | 1(0.21) |  |
| Elevated blood glucose | 2(1.25) |  | 1(0.63) | 1(0.63) | 2(0.63) |  | 4(0.83) |  |
| Various nervous system disorders | 2(1.25) |  | 1(0.63) | 1(0.63) | 2(0.63) |  | 4(0.83) |  |
| Embolic cerebral infarction | 2(1.25) |  | 1(0.63) | 1(0.63) | 2(0.63) |  | 4(0.83) |  |
| Injuries, poisoning and operational complications | 0(0.00) |  | 0(0.00) | 1(0.63) | 1(0.31) |  | 1(0.21) |  |
| Femoral fracture | 0(0.00) |  | 0(0.00) | 1(0.63) | 1(0.31) |  | 1(0.21) |  |
| Musculoskeletal and connective tissue diseases | 2(1.25) |  | 1(0.63) | 1(0.63) | 2(0.63) |  | 4(0.83) |  |
| Osteophyte | 1(0.63) |  | 1(0.63) | 0(0.00) | 1(0.31) |  | 2(0.42) |  |
| Osteonecrosis | 0(0.00) |  | 0(0.00) | 1(0.63) | 1(0.31) |  | 1(0.21) |  |
| Protrusion of intervertebral disc | 1(0.63) |  | 0(0.00) | 0(0.00) | 0(0.00) |  | 1(0.21) |  |
| Surgical and medical procedures | 5(3.13) |  | 3(1.88) | 5(3.13) | 8(2.50) |  | 13(2.71) |  |
| Cancer surgery | 0(0.00) |  | 1(0.63) | 0(0.00) | 1(0.31) |  | 1(0.21) |  |
| Bladder operation | 0(0.00) |  | 1(0.63) | 0(0.00) | 1(0.31) |  | 1(0.21) |  |
| Gallbladder operation | 1(0.63) |  | 0(0.00) | 0(0.00) | 0(0.00) |  | 1(0.21) |  |
| Lung operation | 0(0.00) |  | 1(0.63) | 1(0.63) | 2(0.63) |  | 2(0.42) |  |
| Bone operation | 0(0.00) |  | 0(0.00) | 1(0.63) | 1(0.31) |  | 1(0.21) |  |
| Spinal surgery | 1(0.63) |  | 0(0.00) | 0(0.00) | 0(0.00) |  | 1(0.21) |  |
| Thyroid operation | 0(0.00) |  | 0(0.00) | 2(1.25) | 2(0.63) |  | 2(0.42) |  |
| Vocal cord operation | 1(0.63) |  | 1(0.63) | 0(0.00) | 1(0.31) |  | 2(0.42) |  |
| Esophageal surgery | 0(0.00) |  | 0(0.00) | 1(0.63) | 1(0.31) |  | 1(0.21) |  |
| Cardiac surgery | 2(1.25) |  | 0(0.00) | 0(0.00) | 0(0.00) |  | 2(0.42) |  |
| Various congenital familial hereditary diseases | 1(0.63) |  | 1(0.63) | 0(0.00) | 1(0.31) |  | 2(0.42) |  |
| Limb reduction defect | 1(0.63) |  | 1(0.63) | 0(0.00) | 1(0.31) |  | 2(0.42) |  |
| Respiratory, thoracic and mediastinal disorders | 2(1.25) |  | 3(1.88) | 4(2.50) | 7(2.19) |  | 9(1.88) |  |
| Pulmonary mass | 0(0.00) |  | 1(0.63) | 0(0.00) | 1(0.31) |  | 1(0.21) |  |
| Chronic bronchitis | 1(0.63) |  | 1(0.63) | 2(1.25) | 3(0.94) |  | 4(0.83) |  |
| Chronic obstructive pulmonary disease | 0(0.00) |  | 1(0.63) | 1(0.63) | 2(0.63) |  | 2(0.42) |  |
| Asthma | 0(0.00) |  | 0(0.00) | 1(0.63) | 1(0.31) |  | 1(0.21) |  |
| Bronchiectasis | 1(0.63) |  | 0(0.00) | 0(0.00) | 0(0.00) |  | 1(0.21) |  |
| Benign, malignant and unspecified neoplasms | 1(0.63) |  | 3(1.88) | 2(1.25) | 5(1.56) |  | 6(1.25) |  |
| Bladder cancer | 0(0.00) |  | 1(0.63) | 0(0.00) | 1(0.31) |  | 1(0.21) |  |
| Malignant neoplasm | 0(0.00) |  | 1(0.63) | 0(0.00) | 1(0.31) |  | 1(0.21) |  |
| Malignant lung neoplasm | 0(0.00) |  | 0(0.00) | 1(0.63) | 1(0.31) |  | 1(0.21) |  |
| Cervix carcinoma | 0(0.00) |  | 1(0.63) | 0(0.00) | 1(0.31) |  | 1(0.21) |  |
| Laryngeal neoplasm | 1(0.63) |  | 0(0.00) | 0(0.00) | 0(0.00) |  | 1(0.21) |  |
| Oesophageal carcinoma | 0(0.00) |  | 1(0.63) | 0(0.00) | 1(0.31) |  | 1(0.21) |  |
| Metrocarcinoma | 0(0.00) |  | 0(0.00) | 1(0.63) | 1(0.31) |  | 1(0.21) |  |
| Endocrine system diseases | 0(0.00) |  | 1(0.63) | 1(0.63) | 2(0.63) |  | 2(0.42) |  |
| Hyperthyroidism | 0(0.00) |  | 1(0.63) | 0(0.00) | 1(0.31) |  | 1(0.21) |  |
| Thyroid mass | 0(0.00) |  | 0(0.00) | 1(0.63) | 1(0.31) |  | 1(0.21) |  |
| Skin and subcutaneous tissue disorders | 0(0.00) |  | 1(0.63) | 0(0.00) | 1(0.31) |  | 1(0.21) |  |
| Eczema | 0(0.00) |  | 1(0.63) | 0(0.00) | 1(0.31) |  | 1(0.21) |  |
| Reproductive system and breast diseases | 1(0.63) |  | 0(0.00) | 0(0.00) | 0(0.00) |  | 1(0.21) |  |
| Prostatic disorder | 1(0.63) |  | 0(0.00) | 0(0.00) | 0(0.00) |  | 1(0.21) |  |
| Gastrointestinal disorders | 3(1.88) |  | 1(0.63) | 1(0.63) | 2(0.63) |  | 5(1.04) |  |
| Chronic gastritis | 2(1.25) |  | 1(0.63) | 1(0.63) | 2(0.63) |  | 4(0.83) |  |
| Increased frequency of defecation | 1(0.63) |  | 0(0.00) | 0(0.00) | 0(0.00) |  | 1(0.21) |  |
| Cardiac disorders | 1(0.63) |  | 6(3.75) | 2(1.25) | 8(2.50) |  | 9(1.88) |  |
| Rheumatic heart disease | 0(0.00) |  | 4(2.50) | 1(0.63) | 5(1.56) |  | 5(1.04) |  |
| Coronary artery disease | 0(0.00) |  | 0(0.00) | 1(0.63) | 1(0.31) |  | 1(0.21) |  |
| Chronic cardiac failure | 0(0.00) |  | 1(0.63) | 0(0.00) | 1(0.31) |  | 1(0.21) |  |
| Cardiac hypertrophy | 0(0.00) |  | 1(0.63) | 0(0.00) | 1(0.31) |  | 1(0.21) |  |
| Cardiac disease | 1(0.63) |  | 1(0.63) | 0(0.00) | 1(0.31) |  | 2(0.42) |  |


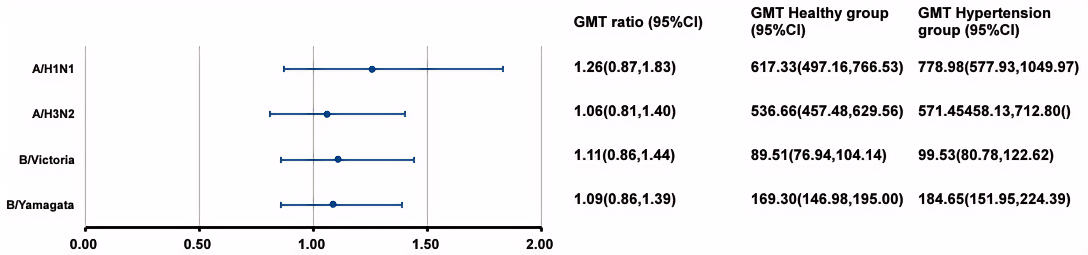


**Figure S1.** Post-vaccination GMTs and GMT ratios for HI antibodies against four influenza virus subtypes(Hypertension group). At 28 days after IIV4 vaccination, the HI antibody GMTs, GMT ratios(Hypertension group/Healthy group), and 95%CIs were calculated. GMT: geometric mean titer; HI: hemagglutination inhibition; IIV4: inactivated quadrivalent influenza vaccine; CI: confidence interval.

Table S2. Post-vaccination antibody levels of four influenza virus strains( Hypertension group ).

|  |  | Healthy group | |  | Hypertension group | |  | Total | | *p*-value |
| --- | --- | --- | --- | --- | --- | --- | --- | --- | --- | --- |
|  |  | (N= 247) | |  | (N=130) | |  | (N=377) | |  |
| Subtype |  | value | 95%CI |  | value | 95%CI |  | value | 95%CI |  |
| A/H1N1 | SPR, %(n) | 90.69(224) | 86.36,94.01 |  | 94.62(123) | 89.22,97.81 |  | 92.04(347) | 88.83,94.57 | 0.1805 |
|  | SCR, %(n) | 89.07(220) | 84.50,92.67 |  | 93.85(122) | 88.23,97.31 |  | 90.72(342) | 87.33,93.45 | 0.1287 |
|  | GMFR | 50.14 | 40.19,62.54 |  | 61.33 | 45.53,82.61 |  | 53.74 | 45.02,64.15 | 0.2880 |
| A/H3N2 | SPR, %(n) | 97.98(242) | 95.34,99.34 |  | 99.23(129) | 95.79,99.98 |  | 98.41(371) | 96.57,99.41 | 0.6688 |
|  | SCR, %(n) | 76.11(188) | 70.30,81.29 |  | 73.08(95) | 64.60,80.48 |  | 75.07(283) | 70.38,79.35 | 0.5172 |
|  | GMFR | 8.56 | 7.14,10.26 |  | 7.19 | 5.64,9.17 |  | 8.06 | 6.97,9.32 | 0.2629 |
| B/Victoria | SPR, %(n) | 82.59(204) | 77.28,87.11 |  | 86.15(112) | 79.00,91.58 |  | 83.82(316) | 79.71,87.39 | 0.3719 |
|  | SCR, %(n) | 72.47(179) | 66.45,77.94 |  | 73.85(96) | 65.42,81.16 |  | 72.94(275) | 68.16,77.37 | 0.7749 |
|  | GMFR | 6.80 | 5.80,7.97 |  | 7.19 | 5.75,9.00 |  | 6.93 | 6.09,7.89 | 0.6851 |
| B/Yamagata | SPR, %(n) | 94.33(233) | 90.67,96.87 |  | 95.38(124) | 90.22,98.29 |  | 94.69(357) | 91.93,96.73 | 0.6647 |
|  | SCR, %(n) | 70.85(175) | 64.75,76.44 |  | 70.00(91) | 61.34,77.72 |  | 70.56(266) | 65.67,75.11 | 0.8633 |
|  | GMFR | 5.18 | 4.44,6.04 |  | 5.87 | 4.72,7.30 |  | 5.41 | 4.77,6.13 | 0.3480 |

SPR: seroprotection rate (≥1:40); SCR: seroconversion rate; GMFR: geometric mean fold rise.


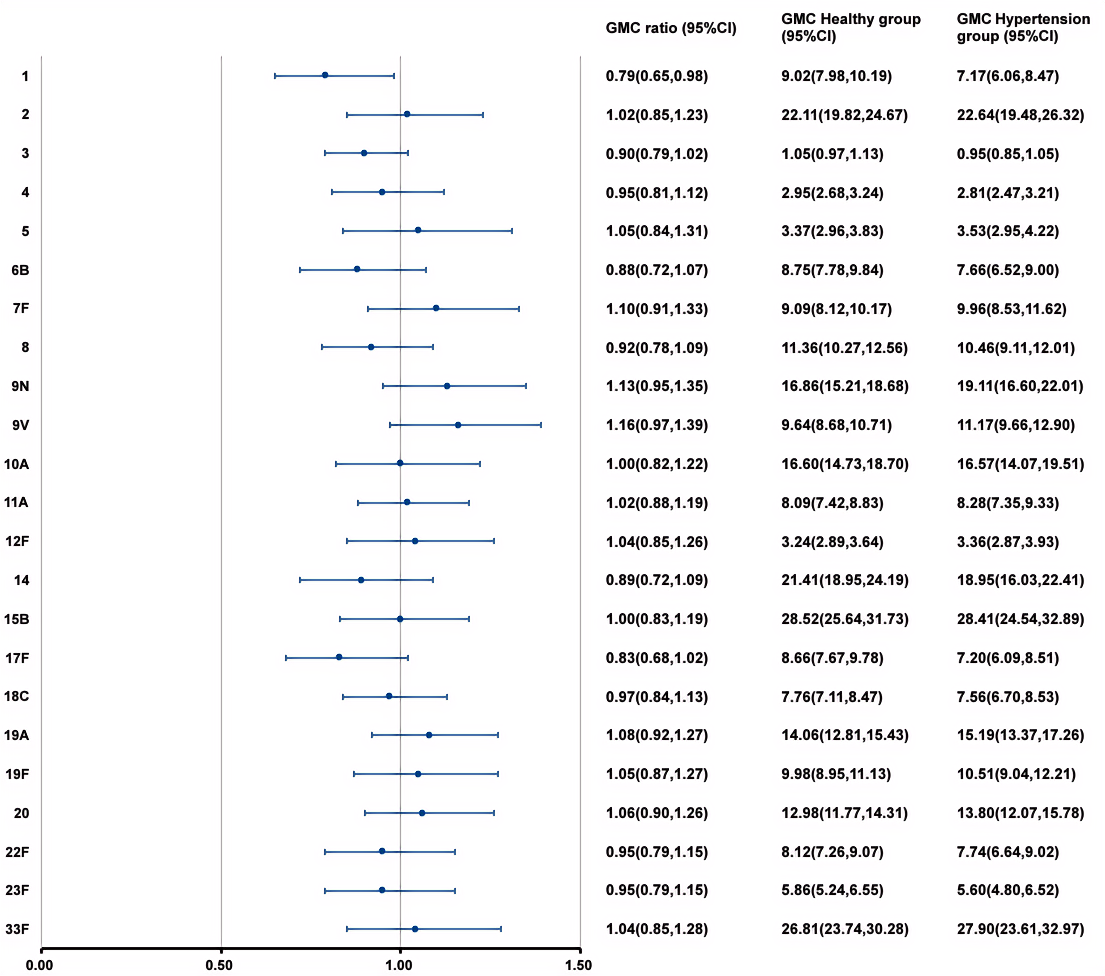


**Figure S2.** Post-vaccination GMCs and GMC ratios for pneumococcal antibodies of 23 serotypes(Hypertension group). At 28 days after PPSV23 vaccination, antibody GMCs, GMC ratios (Hypertension group/Healthy group) and 95% CIs were calculated. GMC: geometric mean concentration; PPSV: pneumococcal polysaccharide vaccine; CI: confidence interval.

Table S3. Post-vaccination antibody levels of 23 pneumococcal serotypes( Hypertension group ).

|  |  | Healthy group | |  | Hypertension group | |  | Total | | *p*-value |
| --- | --- | --- | --- | --- | --- | --- | --- | --- | --- | --- |
|  |  | (N=245) | |  | (N=130) | |  | (N=375) | |  |
| Serotype |  | value | 95%CI |  | value | 95%CI |  | value | 95%CI |  |
| 1 | GMFR | 7.85 | 6.90,8.94 |  | 6.17 | 5.28,7.21 |  | 7.22 | 6.53,7.99 | 0.0248 |
|  | 2-fold increase rate, %(n) | 91.43(224) | 87.20,94.62 |  | 90.77(118) | 84.43,95.14 |  | 91.20(342) | 87.86,93.87 | 0.8302 |
| 2 | GMFR | 7.16 | 6.37,8.04 |  | 7.42 | 6.28,8.76 |  | 7.25 | 6.59,7.97 | 0.7236 |
|  | 2-fold increase rate, %(n) | 93.47(229) | 89.61,96.22 |  | 92.31(120) | 86.31,96.25 |  | 93.07(349) | 90.01,95.42 | 0.6734 |
| 3 | GMFR | 2.26 | 2.07,2.47 |  | 2.21 | 1.98,2.45 |  | 2.24 | 2.10,2.40 | 0.7224 |
|  | 2-fold increase rate, %(n) | 44.49(109) | 38.16,50.95 |  | 54.62(71) | 45.65,63.36 |  | 48.00(180) | 42.84,53.19 | 0.0618 |
| 4 | GMFR | 4.40 | 3.97,4.87 |  | 4.21 | 3.71,4.79 |  | 4.33 | 4.00,4.69 | 0.6130 |
|  | 2-fold increase rate, %(n) | 83.27(204) | 77.99,87.72 |  | 83.08(108) | 75.51,89.08 |  | 83.20(312) | 79.02,86.84 | 0.9630 |
| 5 | GMFR | 7.23 | 6.33,8.27 |  | 7.56 | 6.36,8.98 |  | 7.35 | 6.61,8.16 | 0.6950 |
|  | 2-fold increase rate, %(n) | 89.80(220) | 85.31,93.29 |  | 89.23(116) | 82.59,93.99 |  | 89.60(336) | 86.06,92.50 | 0.8645 |
| 6B | GMFR | 6.99 | 6.19,7.90 |  | 6.17 | 5.21,7.30 |  | 6.69 | 6.07,7.39 | 0.2345 |
|  | 2-fold increase rate, %(n) | 91.43(224) | 87.20,94.62 |  | 86.15(112) | 79.00,91.58 |  | 89.60(336) | 86.06,92.50 | 0.1113 |
| 7F | GMFR | 7.07 | 6.31,7.92 |  | 7.69 | 6.53,9.07 |  | 7.28 | 6.63,7.99 | 0.3999 |
|  | 2-fold increase rate, %(n) | 91.84(225) | 87.67,94.94 |  | 94.62(123) | 89.22,97.81 |  | 92.80(348) | 89.70,95.20 | 0.3218 |
| 8 | GMFR | 5.81 | 5.19,6.51 |  | 5.74 | 4.95,6.65 |  | 5.78 | 5.29,6.33 | 0.8917 |
|  | 2-fold increase rate, %(n) | 88.16(216) | 83.44,91.93 |  | 91.54(119) | 85.36,95.70 |  | 89.33(335) | 85.76,92.27 | 0.3136 |
| 9N | GMFR | 5.99 | 5.37,6.68 |  | 7.35 | 6.17,8.76 |  | 6.43 | 5.85,7.06 | 0.0511 |
|  | 2-fold increase rate, %(n) | 86.53(212) | 81.61,90.54 |  | 90.00(117) | 83.51,94.57 |  | 87.73(329) | 83.98,90.88 | 0.3297 |
| 9V | GMFR | 4.84 | 4.34,5.40 |  | 5.82 | 4.93,6.86 |  | 5.16 | 4.71,5.65 | 0.0598 |
|  | 2-fold increase rate, %(n) | 86.12(211) | 81.15,90.19 |  | 83.85(109) | 76.37,89.71 |  | 85.33(320) | 81.34,88.76 | 0.5532 |
| 10A | GMFR | 7.80 | 6.90,8.80 |  | 7.93 | 6.74,9.32 |  | 7.84 | 7.12,8.64 | 0.8719 |
|  | 2-fold increase rate, %(n) | 91.02(223) | 86.72,94.29 |  | 93.08(121) | 87.26,96.79 |  | 91.73(344) | 88.47,94.31 | 0.4913 |
| 11A | GMFR | 3.19 | 2.90,3.51 |  | 3.31 | 2.91,3.78 |  | 3.23 | 3.00,3.49 | 0.6544 |
|  | 2-fold increase rate, %(n) | 69.80(171) | 63.63,75.48 |  | 69.23(90) | 60.54,77.02 |  | 69.60(261) | 64.67,74.22 | 0.9098 |
| 12F | GMFR | 3.73 | 3.29,4.23 |  | 3.91 | 3.29,4.65 |  | 3.79 | 3.43,4.19 | 0.6623 |
|  | 2-fold increase rate, %(n) | 72.24(177) | 66.19,77.76 |  | 79.23(103) | 71.24,85.84 |  | 74.67(280) | 69.95,78.99 | 0.1388 |
| 14 | GMFR | 4.16 | 3.63,4.77 |  | 3.68 | 3.12,4.36 |  | 3.99 | 3.59,4.44 | 0.2820 |
|  | 2-fold increase rate, %(n) | 68.57(168) | 62.35,74.33 |  | 67.69(88) | 58.93,75.63 |  | 68.27(256) | 63.29,72.95 | 0.8618 |
| 15B | GMFR | 6.15 | 5.47,6.91 |  | 6.68 | 5.65,7.89 |  | 6.32 | 5.75,6.96 | 0.4183 |
|  | 2-fold increase rate, %(n) | 87.76(215) | 82.98,91.58 |  | 86.15(112) | 79.00,91.58 |  | 87.20(327) | 83.39,90.41 | 0.6587 |
| 17F | GMFR | 6.63 | 5.80,7.59 |  | 5.77 | 4.89,6.83 |  | 6.32 | 5.69,7.02 | 0.2161 |
|  | 2-fold increase rate, %(n) | 85.31(209) | 80.24,89.49 |  | 83.08(108) | 75.51,89.08 |  | 84.53(317) | 80.47,88.04 | 0.5699 |
| 18C | GMFR | 3.57 | 3.24,3.93 |  | 3.68 | 3.27,4.14 |  | 3.61 | 3.34,3.89 | 0.7120 |
|  | 2-fold increase rate, %(n) | 77.14(189) | 71.37,82.25 |  | 81.54(106) | 73.79,87.80 |  | 78.67(295) | 74.17,82.71 | 0.3227 |
| 19A | GMFR | 2.79 | 2.54,3.07 |  | 3.05 | 2.67,3.47 |  | 2.88 | 2.67,3.10 | 0.2860 |
|  | 2-fold increase rate, %(n) | 64.08(157) | 57.73,70.09 |  | 64.62(84) | 55.75,72.80 |  | 64.27(241) | 59.19,69.12 | 0.9182 |
| 19F | GMFR | 4.85 | 4.33,5.42 |  | 5.11 | 4.39,5.95 |  | 4.94 | 4.51,5.40 | 0.5770 |
|  | 2-fold increase rate, %(n) | 82.86(203) | 77.54,87.36 |  | 82.31(107) | 74.65,88.44 |  | 82.67(310) | 78.45,86.36 | 0.8936 |
| 20 | GMFR | 3.16 | 2.87,3.49 |  | 3.39 | 2.96,3.87 |  | 3.24 | 2.99,3.50 | 0.4169 |
|  | 2-fold increase rate, %(n) | 66.53(163) | 60.24,72.41 |  | 73.85(96) | 65.42,81.16 |  | 69.07(259) | 64.12,73.71 | 0.1447 |
| 22F | GMFR | 4.80 | 4.27,5.39 |  | 4.77 | 4.11,5.53 |  | 4.79 | 4.37,5.25 | 0.9493 |
|  | 2-fold increase rate, %(n) | 81.63(200) | 76.21,86.28 |  | 83.85(109) | 76.37,89.71 |  | 82.40(309) | 78.16,86.12 | 0.5922 |
| 23F | GMFR | 5.32 | 4.75,5.95 |  | 5.34 | 4.48,6.36 |  | 5.33 | 4.84,5.86 | 0.9727 |
|  | 2-fold increase rate, %(n) | 85.31(209) | 80.24,89.49 |  | 84.62(110) | 77.24,90.34 |  | 85.07(319) | 81.05,88.52 | 0.8582 |
| 33F | GMFR | 8.99 | 7.94,10.18 |  | 9.42 | 7.87,11.26 |  | 9.14 | 8.25,10.11 | 0.6694 |
|  | 2-fold increase rate, %(n) | 93.47(229) | 89.61,96.22 |  | 93.08(121) | 87.26,96.79 |  | 93.33(350) | 90.32,95.64 | 0.8847 |

GMFR: geometric mean fold rise.
